# DNA Methylation Profiling Reveals the Change of Inflammation-Associated *ZC3H12D* in Leukoaraiosis

**DOI:** 10.3389/fnagi.2018.00143

**Published:** 2018-05-23

**Authors:** Wen-Qing Huang, Ke-Hui Yi, Zhi Li, Han Wang, Ming-Li Li, Liang-Liang Cai, Hui-Nuan Lin, Qing Lin, Chi-Meng Tzeng

**Affiliations:** ^1^Translational Medicine Research Center, School of Pharmaceutical Sciences, Xiamen University, Xiamen, China; ^2^Key Laboratory for Cancer T-Cell Theranostics and Clinical Translation, Xiamen University, Fujian, China; ^3^Department of Physiology, Zhongshan School of Medicine, Sun Yat-sen University, Guangzhou, China; ^4^Department of Neurology, The First Affiliated Hospital of Xiamen University, Xiamen, China; ^5^Department of Neurology, The First Clinical College of Fujian Medical University, Fuzhou, China; ^6^INNOVA Cell: TDx/Clinics and TRANSLA Health Group, Yangzhou, China; ^7^College of Pharmaceutical Sciences, Nanjing Tech University, Nanjing, China; ^8^Jiansu Provincial Institute of Translation Medicine and Women-Child Health Care Hospital, Nanjing Medical University, Nanjing, China

**Keywords:** demyelination, leukoaraiosis (LA), methylation, neuroinflammation, white matter lesions (WML), ZC3H12D

## Abstract

Leukoaraiosis (LA) is neuroimaging abnormalities of the cerebral white matter in elderly people. However, the molecular mechanisms underlying the cerebral white matter lesions remain unclear. Here, we reported an epigenetic basis and potential pathogenesis for this complex illness. 317 differentially methylated genes were identified to distinguish the mechanism of occurrence and progression of LA. Gene-Ontology pathway analysis highlighted that those genes with epigenetic changes are mostly involved in four major signaling pathways including inflammation and immune response-associated processes (antigen processing and presentation, T cell costimulation and interferon-γ-mediated signaling pathway), synapse assembly, synaptic transmission and cell adhesion. Moreover, immune response seems to be specific to LA occurrence and subsequent disruption of nervous system functions could drive the progression of LA. The significant change of inflammation-associated *ZC3H12D* in promoter methylation and mRNA expression was implicated in the occurrence of LA, suggesting its potential functions in the molecular mechanism of LA. Our results suggested that inflammation-associated signaling pathways were involved in the pathogenesis of LA and *ZC3H12D* may contribute to such inflammatory process underlying LA, and further echoed it as a neuroinflammatory disorder in central nervous system (CNS).

## Introduction

Leukoaraiosis (LA), also known as white matter lesions (WML) and white matter hyperintensities (WMH), is a common finding on fluid-attenuated inversion recovery-magnetic resonance imaging (FLAIR-MRI) of the brain in elderly people beginning in middle age, with prevalence from 50 to 100% worldwide (Hachinski et al., 1987; Kim et al., 2008; Xiong and Mok, 2011). Although LA often remains asymptomatic, it is not considered to be benign but increasingly shown to be strongly related to a host of poor clinical outcomes, and increase the risk for disability (gait disturbance and falls), cognitive function decline, dementia, depression and stroke, as well as the overall morbidity and mortality (Kim et al., 2008; Debette and Markus, 2010). Pathologically, LA is only characterized by myelin pallor, tissue rarefaction associated with loss of myelin and axons, patchy demyelination, mild gliosis and denudation of the ependyma in regions of white matter hyperintensity, but the mechanisms leading to the pathophysiology and neuroimaging abnormalities of the cerebral white matter remain unclear (Brun and Englund, 1986; O'Sullivan, 2008).

Up to date, age and hypertension are the only generally accepted and most importantly established risk factors for LA (Grueter and Schulz, 2012). Previous genetic studies showed that the heritability of LA was up to 80% (Carmelli et al., 1998; Atwood et al., 2004; Turner et al., 2004). Moreover, a large account of gene loci were identified to contribute to the increased risk of LA during the past decade (Carmelli et al., 1998; Turner et al., 2004; Smith et al., 2009; Fornage et al., 2011). They include not only *TRIM65, TRIM47*, and *WBP2* on genome-wide significant Chr17q25 locus for WMH burden that was identified in the genome-wide association study (GWAS) of LA in European descent (Grueter and Schulz, 2012), but also those risk factors which were revealed by candidate gene association studies, such as aging-related gene (e.g., *APOE*), hypertension-associated genes (e.g., *ACE, AGT, AGTR1*), homocysteine metabolism gene (*MTHFR*), oxidative stress-related genes (e.g., *PON1, NOS3*) and inflammation-associated genes (e.g., *IL6, IL5RA, NR3C1, PTGS2, AQP4*) (Duan et al., 2012; Shan et al., 2013; Yadav et al., 2014; Lin et al., 2015). Recently, a multi-ethnic genome-wide association study identified four novel genetic loci including Chr10q24 (e.g., *SH3PXD2A*), Chr2p21 (e.g., *HAAO*), Chr2p16 (e.g., *EFEMP1*) and Chr1q22 (e.g., *PMF1*) (Verhaaren et al., 2015). Additionally, together with microarray RNA expression analysis of cerebral white matter lesions tissues, multiple whole blood expression profiles revealed large numbers of up-/down-regulated genes in LA (Simpson et al., 2009; Xu et al., 2010). These evidences support the multifactorial nature of LA and genetic factors seem to play important roles in LA. However, the functions of these risk genes in the pathogenesis of LA are poorly understood. Moreover, it is still unclear that whether other unknown risk factors (such as epigenetic factors) also participated in the pathophysiology of LA. It's therefore urgently needed to explore the pathogenesis and find efficiently potential strategies for its prevention and management.

Together with multiple epidemiological studies on LA and a lot of review literature about LA, our previous studies indicate LA as a complex illness which may be driven by both genetic and environmental factors, such as smoking and drinking. In this study, we aimed to identify an epigenetic basis for this complex trait and to decipher the pathogenic genes and signaling pathways during the occurrence and/or progression of LA in an innovative case–control study of LA through genome-scale methylation analysis in Chinese population.

## Subjects and methods

### Study participants

**Figure 1:**
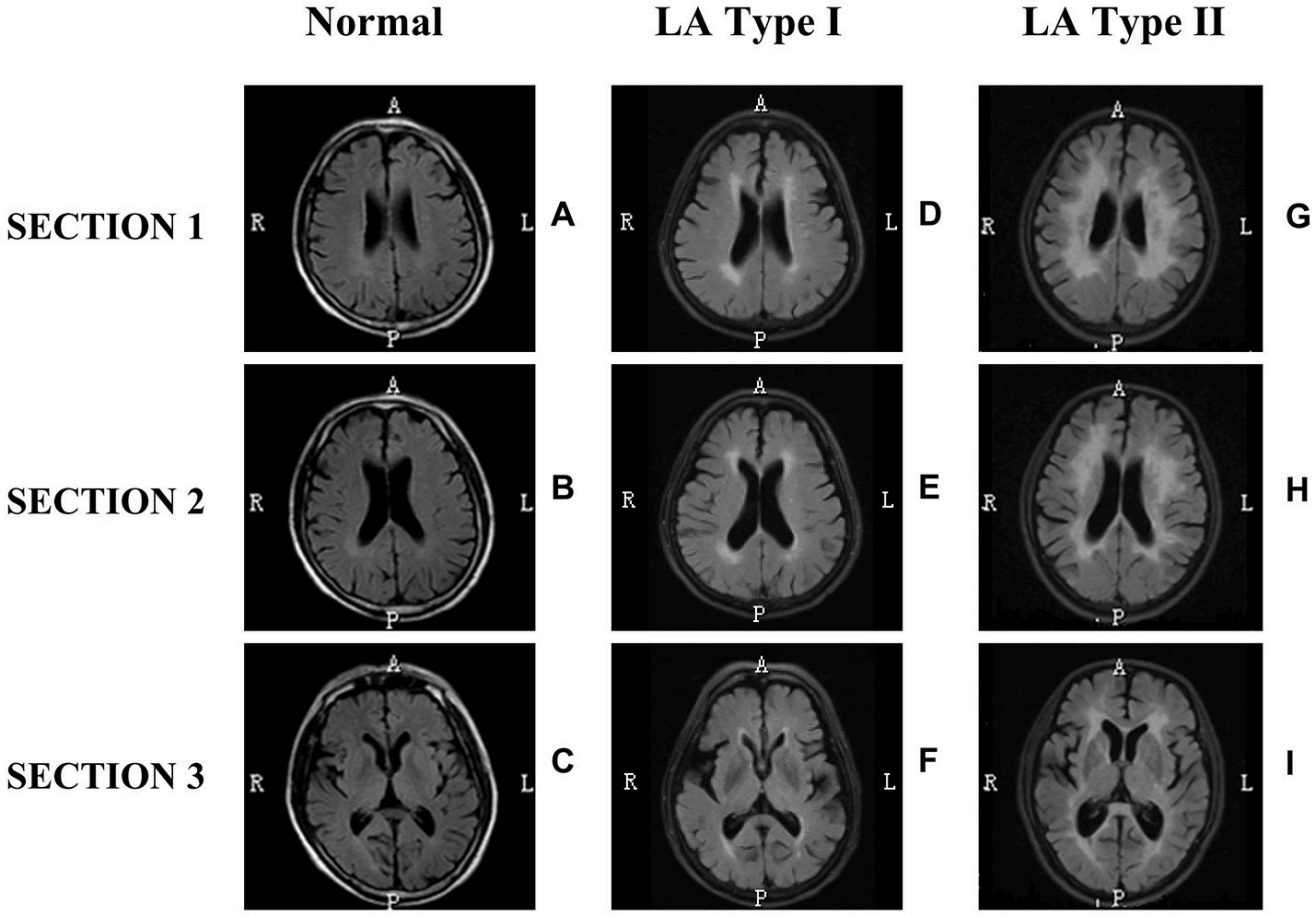
Neuroimaging classification of leukoaraiosis (LA). LA was defined according to the systematical assessment of white matter lesions on three section (1, 2, 3) of neuroimaging of subjects **(D,E,F or G,H,I)**. LA type I **(D,E,F)** and LA type II **(G,H,I)** showed white matter hyperintensity on all the three sections of T_2_-weighted FLAIR MRI scans, but not on Normal **(A–C)**. Sections 1, 2, 3 represent the centrum semiovale section, lateral ventricles section, and internal capsule section, respectively. The first column shows few white matter lesions on all three sections of MRI imaging. The second column shows white matter changes including a pencil-thin lining or smooth halo along the side of the lateral ventricles, small periventricular caps around the frontal horns of the lateral ventricles, with early confluent lesions. The third column shows irregular periventricular lesions, largely extending periventricular caps, and severely confluent abnormalities. In addition, this classification scheme was proposed to divide LA-associated neuroimaging phenomenon into three broad categories: Normal **(A–C)**, LA type I **(D–F)** and LA type II **(G–I)**, based on both the severity of white matter lesions and new definition of LA. This classification method of LA neglected the neuroimaging distinctions between periventricular WMLs (PVWMLs) and deep/subcortical WMLs (DWMLs) and reduced the heterogeneity for helping clinicians and neuroscientists easily to diagnose LA and to evaluate severity precisely. It's helpful for scientists to decipher the pathogenic mechanism and clinically functional consequence properly.

To accrue a cohort of patients with LA and disease controls, we initially assessed all patients seen at the department of neurology in the first affiliated hospital of Xiamen university, with clinical brain T2-weighted MRI between 2012 and 2013. Before the case–control study, we summarized a new neuroimaging definition for LA based on the assessment of white matter lesions on three sections of brain MRI, and further proposed a new classification method to classify LA into two subgroups: LA Type I and LA Type II (Figure 1). Inclusion criteria of study participants including LA and disease controls was established based on the established re-definition and classification scheme of LA in our laboratory. Eligibility criteria included: (a) age from 60 to 80, (b) none, mild or severe changes in cerebral subcortical white matter on brain MRI, and (c) sex: male. Exclusion criteria were hypertension, diabetes, hyperhomocysteinemia, dementia, Parkinson's disease or Alzheimer's disease, as well as history of smoking. In addition, subjects were also excluded with evidence of intracerebral or subarachnoid hemorrhage, intracranial infection, malignant tumor, toxic encephalopathy, multiple sclerosis or hydrocephalus. Finally, a total of 9 subjects were recruited for genome-wide methylation analysis and divided into three subgroups: Normal (controls without LA, *n* = 3), LA Type I (*n* = 3), and LA Type II (*n* = 3) (Table 1). Additionally, 9 independent subjects only meeting eligibility criteria of age and neuroimaging definition were also recruited for subsequent validation of methylation microarray findings. This study was approved by Xiamen ethical committee, and all study subjects provided written informed consent.

**Table 1:** Clinical details of study subjects.

**Subjects**	**Label**	**Age (years old)**	**Systolic/diastolic blood pressure (mm Hg)**	**Glucose(mmol/L)**	**Smoking**	**Drinking**	**LDL-C (mmol/L)**	**HCY (nmol/L)**	**MRI-imagin (LA/Control)**
1	B1BD03	70–75	145/90	5.47	–	–	3.14	8.55	Normal
2	B1BD07	70–75	133/100	4.05	–	–	3.65	18.93	
3	B1BD11	70–75	105/71	5.20	+	–	2.95	14.77	
4	B1BE03	60–65	142/80	5.70	+	–	2.89	10.29	LA Type I
5	B1BE07	60–65	170/90	4.90	+	–	1.83	19.61	
6	B1BE11	70–75	153/79	6.49	–	–	3.95	37.93	
7	B1BF03	60–65	133/80	4.10	+	+	3.13	14.81	LA Type II
8	B1BF07	66–70	127/78	5.43	–	–	2.15	13.17	
9	B1BF11	80–85	116/80	6.60	–	–	2.98	26.95	

*LDL-C, low density lipoprotein cholesterol; HCY, homocysteine; LA, leukoaraiosis*.

### Genomic DNA extraction

Genomic DNA for DNA methylation analysis and subsequent validation of microarray analysis was extracted from frozen whole blood of 9 subjects using MagCore® Genomic DNA Whole Blood Kit (Cat. No: MGB400-04,RBC Bioscience, Taiwan, China) according to the manufacturer's protocol. DNA was quantitated with the NanoDrop®ND-1000 UV–Vis Spectrophotometer (NanoDrop Technologies, Wilmington, DE, USA). Integrity of genomic DNA was evaluated and confirmed by agarose gel electrophoresis and capillary electrophoresis (CE), respectively.

### Illumina 450K-array methylation analysis

High quality DNA was bisulfite converted using the EZ DNA Methylation-Gold™ Kit (Cat. No: D5006, Zymo Research, Orange, CA, USA) according to manufacturer's instructions. Converted DNA was then hybridized to the Illumina Human Methylation450K BeadChip and analyzed following the 450K Methylation array assay procedure (Illumina, San Diego, CA, USA) (www.illumina.com). In order to prevent potential batch effects, 9 genomic DNA samples were randomly assigned assay wells in the same array and they were processed using the same batch of reagents and processing procedure. Hybridization fluorescent signals of each CpG site were read by the Illumina scan system. Raw signal intensities of each probe (without background correction or normalization) were extracted through the Illumina's GenomeStudio® software (version 2011.1; Illumina Inc.). Firstly, for quality control, those probes with a low bead count (<3) and high detection *P*-value (>0.01) and samples with a low success rate (<95%) were removed. Then, raw signal intensities of every sample were individually normalized through internal probe controls and background subtraction. Finally, β-value, a quantitative measure parameter of methylation levels of each CpG sites was then assigned via Illumina GenomeStudio software. This value was calculated from the ratio of fluorescent signals from the methylated alleles to the sum of the signals from the methylated and unmethylated alleles [β = M / (M + U + α) where M and U are methylated and unmethylated signal intensities, respectively. In addition, α is an arbitrary offset (usually 100) intended to stabilize β-values where fluorescent intensities are low (Bibikova et al., 2011). β values ranged from 0 (completely unmethylated, U) to 1 (fully methylated, M) on a continuous scale over the different CpG sites studied. To determine differential methylation patterns between groups for all group comparisons, Δβ was defined as the relative difference in methylation levels between the CpG site of the experimental and control specimens (Bibikova et al., 2011). We considered a probe as differentially methylated sites if the absolute value of the difference between robust β-values medians in samples of each group is higher than 0.17 (|Δβ|≥ 0.17). This threshold, representing approximately a difference in DNA methylation levels of 17%, corresponds to the recommended difference between samples that can be detected with 99% confidence (Bibikova et al., 2006, 2011). For the differential methylation analysis module, the parameter DiffScore was also used to assess the significance of the difference between methylation levels of a given CpG locus in two groups: DiffScore = 10^*^sgn (β_cond_ – β_ref_) log10 (*p*), where β_cond_ is the β-value of an analyzed CpG site, β_ref_ is the β-value of the control CpG site, and *p* is the probability of flurescence signal from the analyzed sequence to differ from the control signal (Bibikova et al., 2006). A negative DiffScore value reflects a hypomethylated state of the CpG site as compared to the control and a positive value reflects a hypermethylated state. DiffScore>13 correspond to *p*-values of 0.05. Final results were visualized by means of a heat-map using the genesis program (http://genome.tugraz.at) and three normal controls were included for comparison purposes. A CpG site with |Δβ|≥ 0.17 and |DiffScore|>13, was considered to be statistically significant. β-values with assigned detection *p* > 0.01 were treated as missing data. CpG sites with more than 1% missing data (β-values) across all samples were discarded. Finally, we plotted the -log10 (*p*-values) from the robust linear regression for 485577 CpG sites across the genome in 9 participants. All statistical analyses were performed using the Illumina GenomeStudio software (version 2011.1; Illumina Inc).

### Pyrosequencing

To validate the data from the methylation array, those significantly methylated CpG sites located in key genes (e.g., *ZC3H12D, WDR41, BRUNOL4*, and *LHX3*) were chosen for DNA methylation quantitation via pyrosequencing analysis. Firstly, 1 μg of genomic DNA was bisulfite treated using the Epitect bisulfite kit (Cat. No: 59110, Qiagen, Germany) followed by PCR amplification and denaturation according to standard protocols (Qiagen, Germany). Secondly, quantitative methylation analysis was then performed on the PyroMark Q24 instrument (Qiagen, Germany). Epitect fully converted and unconverted DNA controls were included for analysis (Qiagen, Germany). Details of the PCR and sequencing primers for pyrosequencing in each gene are seen in Supplemental Information (Supplemental Table S1).

### Pathway analysis

Gene ontology (GO) analysis of those differentially methylated genes was conducted through the DAVID Bioinformatics Resources 6.8 (http://david.abcc.ncifcrf.gov). Then, the KEGG database was used to identify functional gene groups and ontology terms that were significantly overrepresented. GO terms with *p* < 0.05, and KEGG pathways with *p* < 0.05 were considered significantly enriched. Over-representation of pathways and biological process in the differentially methylated genes was determined with FunNet software (Functional Analysis of Transcriptional Networks).

### Total RNA extraction and quantitative real time PCR (qPCR)

Total RNA of clinical blood and U251 cell line samples were extracted using the TRIzol™ reagent according to TRIZOL method (Cat. No: 15596026, Invitrogen, USA). Quality and integrity of RNA were determined through the NanoDrop®ND-1000 UV–Vis Spectrophotometer (NanoDrop Technologies, Wilmington, USA) and agarose gel electrophoresis. The first-strand cDNA was synthesized from 1 μg of total RNA using SuperScript® III Reverse Transcriptase Kit (Cat. No: 18080044, Invitrogen, USA) following the protocol. All of qPCR reactions were performed through SYBR Green-based methods together with the GoTaq® qPCR Master Mix (Cat. No: A6001, Promega, USA) and primers on Agilent Mx3005P Real-Time PCR system (Agilent, USA). 1 μl cDNA was used in a total volume of 20 μl, with a primer concentration of 200 nM. Every reaction was initiated at 10 min at 95°C, followed by 40 cycles consisting of 15 s denaturation at 95°C, 60 s annealing at 60°C and 30 s extension at 72°C. A final dissociation stage was run to generate a melting curve for verification of amplificated product specificity. The relative mRNA expression levels with respect to the housekeeping gene (*GAPDH*) were measured using the 2^−ΔΔCT^ method. All qPCR experiments were performed in triplicate. Primer pairs were designed with the Primer Premier 5 software (Premier Biosoft International, Palo Alto, CA) and validated by iPAGE.

### Cell culture and DNA demethylation with 5′-aza-2′-deoxycytidine

Human glioma cell line U251 and breast tumor cell line MCF7 were cultured in DMEM/F12 medium (Hyclone™, Thermo) supplemented with 10% fetal bovine serum (FBS) (Gibco, USA), 100 units/mL penicillin, and 100 μg/mL streptomycin at 37°C in a 5%CO_2_ incubator. For the demethylation test, when cells seeded in 6-well plates reached 40% confluence, U251 and MCF7 were treated with 5′-aza-2′-deoxycytidine (5′-Aza) (Cat. No: A3656-5MG, Sigma, St Louis, MO, USA) with 0, 5, 10, and 20 μM final concentration, a well-used methyltransferase inhibitor, respectively. Protein was extracted after 48 h of 5-Aza treatment for the following analysis.

### Protein detection

Total cell extracts were harvested in radioimmunoprecipitation (RIPA) lysis buffer (Cat. No: R0010-100, Solarbio, China) supplemented with protease inhibitors on ice. Protein quantification was measured by Pierce™ BCA protein assay kit (Cat. No: 23227, Thermo Fisher Scientific, USA). Protein detection was conducted through SDS-PAGE and the following western blot according to standard methods. Proteins were separated by 10% SDS-PAGE, and transferred onto PVDF membranes (Millipore Corporation, Billerica MA, USA). Membranes were blocked overnight with 5% non-fat dried milk for 2 h and incubated with anti-ZC3H12D antibody (Cat. No: 24991-1-AP, Proteintech, USA) at 1:5000 dilution; anti-GAPDH antibody (Cat. No: 10494-1-AP, Proteintech, USA) at 1:50,000 dilutions overnight at 4°C. After washing with TBST (10 mM Tris, pH 8.0, 150 mM NaCl, and 0.1% Tween20), the membranes were incubated for 4 h at room temperature with goat anti-rabbit second antibody at 1:20,000. Finally, the protein bands were detected by enhanced chemiluminescence (ECL) with BioSpectrum Gel Imaging System (UVP, USA).

### Statistical analysis

Data on the DNA methylation and mRNA expression levels are reported as means ± standard deviation (SD). *t*-test was used to analyze the statistical significance of DNA methylation and gene expression. All *p*-values were calculated using a two-sided test and a *p*-value of less than 0.05 (*p* < 0.05) was considered statistically significant. All statistical analysis and graph construction were performed using SPSS software version 17.0 (IBM, USA) and GraphPad Prism Software version 5.0 (GraphPad, USA). The hierarchical clustering of gene expression was conducted through RT^2^ Profiler PCR Array Data Analysis Version 3.5 (Qiagen, USA).

## Results

### Aberrant DNA methylation in LA

To gain mechanistic insights into LA, we performed a comprehensive genome-wide DNA methylation profiling analysis of 6 LA blood samples and 3 healthy samples (Normal) using the Illumina Human Methylation450K Array (Bibikova et al., 2006, 2011), and identified 202 CpG sites (including 135 hypermethylated sites and 67 hypomethylated sites) with significant DNA methylation variability between LA and controls (Supplemental Table S2). We further examined the genomic characteristics of the sites identified as differing between LA and Normal (Control), and revealed that a large proportion of those sites were not situated within CpG island which are often located at gene promoter regions and strongly regulated the transcription levels of genes, but located in the gene body. A spatial view of differentially methylated sites mapped across the genome showed that those sites were enriched on the chromosomes 1, 2, 6, and 8, showing a few bias in the distribution across all chromosomes (Supplemental Figure S1A). 51% (103/200 sites) of above differentially methylated sites were identified to locate in non-annotated regions in relation to the CpG island. A regional analysis of those remaining sites in relation to CpG islands noted the majority of sites to be positioned outside of those known annotated CpG islands. More specifically, of the available annotated regions, 31% were located in CpG islands, 26% in N_shores, 26% in S_shores, 12% in N_shelves and 5% in S_shelves (Supplemental Figure S1B). Those CpG sites could also be annotated according to gene functional categories. As shown in Supplemental Figure S1C, most of them were annotated to be in gene body (up to 59%), 200 or 1,500 bp of a transcription start site (TSS) (27%), 5′ untranslated region (UTR) (9%) and 3′UTR (5%). Among those significantly methylated CpG sites in LA, 132 hypermethylated sites and 66 hypomethylated sites were annotated to 68 genes and 34 genes, respectively (Supplemental Figures S1D,E). Hence, these results for the first time showed that aberrant DNA methylation was implicated in leukoaraiosis and epigenetic modification may play a key role in this complex illness.

### Identification of differentially methylated genes during the occurrence and progression of LA

**Figure 2:**
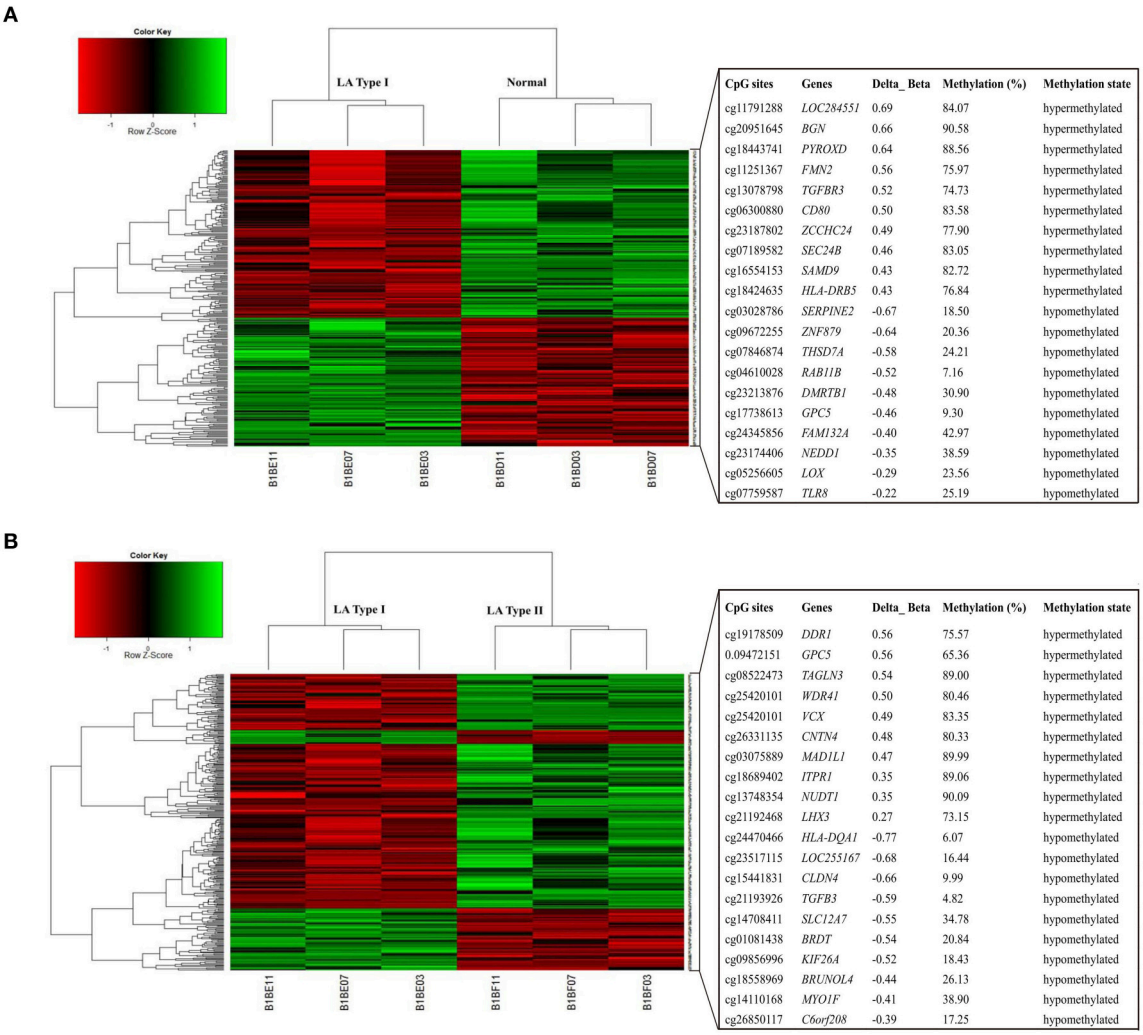
Heatmap of differentially methylated genes in both LA occurrence and progression. The top and low heatmaps demonstrated supervised hierarchical clustering of hypermethylated and hypomethylated genes in the occurrence (LA type I vs. Normal) **(A)** and in progression (LA type II vs. type I) **(B)**, respectively. The green/red color represented a standardized level of DNA methylation both in LA type I (vs. Normal) and in LA type II (vs. LA type I). The list of clustered genes is selectively represented in the right panels. The top and low panels showed the hypermethylated genes and hypomethylated genes in the occurrence **(A)** and progression **(B)** and its methylation level in LA type I **(A)** and LA type II **(B)** respectively.

**Figure 3:**
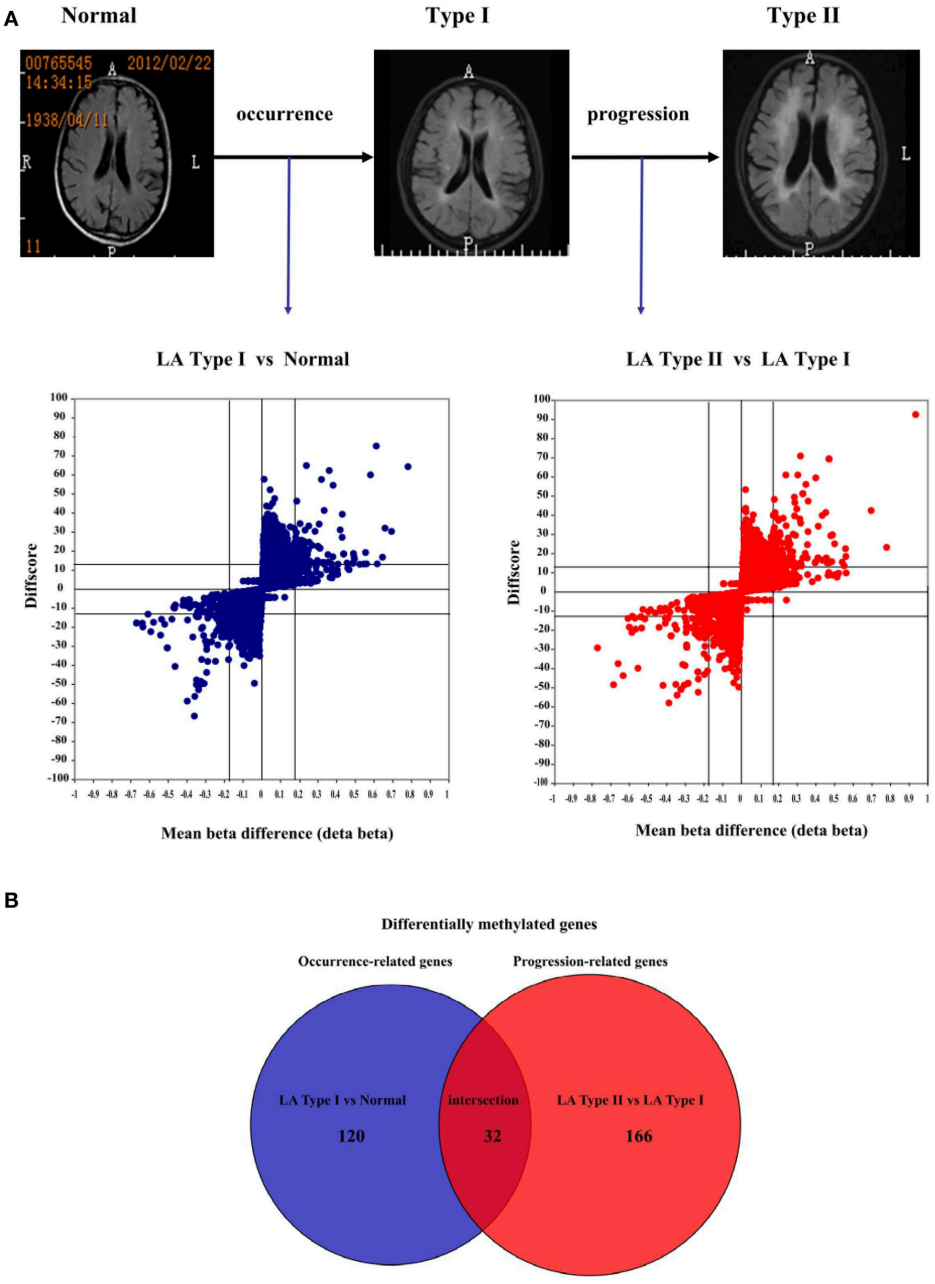
Differential DNA methylation profiles between LA occurrence and progression. **(A)** Putative physiological processes of human brain imaging showing LA occurrence and progression and volcano plots showing statistically significant DNA methylation difference in LA type I vs. Normal and LA type II vs. LA type I. Those significant CpG sites identified in LA type I (vs. Normal) and LA type II (vs. LA type I) are considered to be the LA occurrence-associated sites and LA progression-associated sites, respectively. The X axis represents the mean beta difference (Δβ) which indicates the relative methylation change within groups. The Y axis represents absolute DiffScore value for each significant CpG sites from the two sided *t*-test. The differential methylation cutoff is |Δβ|≥ 0.17 and |DiffScore|>13.The blue/red dot represents the significant CpG sites identified from the comparison of LA type I with Normal and the comparison of LA type II with LA type I, respectively. **(B)** Venn diagram showing highly significant overlapping between differentially methylated genes identified in LA occurrence and those in progression. The number in the blue circle represents the number of differentially methylated genes associated with LA occurrence in the comparison (LA type I vs. Normal). The number in the red circle represents the number of differentially methylated genes associated with LA progression in the comparison (LA type II vs. LA type I). The number of the crimson circle represents the number of overlapping genes across these different comparisons.

To determine the heterogeneity between LA type I and type II, and identify differentially methylated CpG sites in LA occurrence and progression, we obtained the DNA methylome profile of these two subgroups and further performed a locus-by locus DNA methylation analysis for Type I vs. Normal and Type II vs. Type I, respectively (Figure 2). Using our criteria of DiffScore ≥13 and a minimum median β-value difference of 0.17, we screened 269 probes and 232 probes in the occurrence and progression, respectively (Figure 3A). For those differentially methylated probes, we displayed the genomic locations of the occurrence-associated and progression-associated loci and compared the proportions of LA-associated loci for each chromosome using permutation testing to determine if occurrence-associated or progression-associated DNA methylation is specific to certain regions of the genome. This analysis showed that both disease processes have differentially methylated loci located throughout the genome, and revealed an enrichment of occurrence-associated methylated genes on the chromosomes 1, 2, 6, 8, and progression-associated methylated genes on the chromosomes 2, 5, 6, 7, 8, 12 (Supplemental Figure S2A). In addition, we examined the proportion of occurrence-associated methylated loci and progression-associated loci on CpG island and CpG island “shores” respectively, and found higher proportion of loci within CpG island “shores” in the occurrence and equally high proportion of loci within CpG island and CpG island “shores” in the progression (Supplemental Figures S2BC). Interestingly, we also observed that most of 269 loci (56.5%) were hypomethylated in the occurrence, but most of 232 loci (78.4%) were hypermethylated in the progression (Supplemental Figure S2D). Moreover, more aberrant methylated loci distributed in every gene region including gene body, TSS200 and TSS 1500 region, 5′UTR, the first exon of gene (1st exon) and 3′UTR occurred in the progression than in the occurrence (Supplemental Figure S2E). These findings also strongly indicated different epigenetics in these two molecule pathogenesis of LA.

For deciphering the distinct pathogenic genes and/or shared genes in the LA occurrence and progression, the above identified probes were mapped to 320 annotated genes. They consist of 152 genes and 198 genes in the occurrence and progression, respectively. For the occurrence process, 53 of the 152 genes (34.9%) were significantly hypermethylated, whereas 99 (65.1%) were significantly hypomethylated (Figures 3A,B, Supplemental Table S3). For the progression process, 150 of the 198 genes (75.8%) were significantly hypermethylated, whereas 48 (24.2%) were significantly hypomethylated (Figures 3A,B, Supplemental Table S4). Furthermore, we investigated whether this above differential methylation was occurrence-specific or progression-special and found that 32 genes identified in LA type I compared with Normal overlapped those identified in LA type II compared with LA type I, representing shared genes that were differentially methylated in both occurrence and progression (Supplemental Table S5). Intriguingly, we also revealed that 28 of 32 genes (87.5%) were inversely methylated in the occurrence and progression, of which, 6 genes (*HLA-DRB5, HLA-DQB1, TAPT1, TNNI3, ZCCHC12, TNNT1*) hypermethylated in the occurrence were hypomethylated in the progression, whereas 22 genes (*CDYL, CYP1A1, KCNQ1, TUBGCP6, CSTA, P2RX5, CACNA2D4, TAF4, MYOZ3, FMOD, CYHR1, KIFC2, PSTPIP2, OTUD6B, TRANK1, WDR41, GPC5, VCX, RAB11B, HCN2, LHX3, THADA*) hypomethylated in the occurrence were hypermethylated in the progression. Above described, the changes of those genes methylation indicated that they may not mainly contribute to the disease occurrence, but the progression. The remaining four genes were hypermethylated or hypomethylated in both occurrence and progression, including *DDR1, HLA-DQA1, LOC255167, and RAD51L1*. They could not mainly contribute to the disease progression, but the occurrence. However, it's no doubt that other differentially methylated genes identified in LA type I (vs. Normal) and LA type II (vs. LA type I) potentially contributed to the LA occurrence and progression, respectively. On the other hand, to further determine the causing genes of these two processes, we also performed a locus-by locus DNA methylation analysis of LA type II vs. Normal, and screened the differentially methylated genes underlying the complex process from normal brain imaging to white matter hyperintensities (Supplemental Table S6). We next overlapped those genes identified in LA type II (vs. Normal) with those identified in LA type I (vs. Normal) and those in LA type II (vs. LA type I), respectively, and found 11 genes (*BGN, CD80, IGSF21, KIF1B, LOX, RBM47, RESP18, SAMD9, SMTNL2, SNED1, ZC3H12D*) which may specially contribute to the occurrence and 20 genes (*ATP8A2, BRUNOL4, C1orf127, CES1, GIPC2, HECW2, HLA-DRB1, IGLON5, IQSEC3, LOC222699, LOC284379, MAD1L1, OR5A1, RALYL, RFPL2, SLC12A7, TAGLN3, TANC1, TGFB3, ZNF92*) which appeared to contribute to the progression. Thus, these findings strongly indicated different DNA methylation pattern and epigenetic mechanisms implicated in LA occurrence and progression, respectively.

### Validation of DNA-methylation data

**Figure 4:**
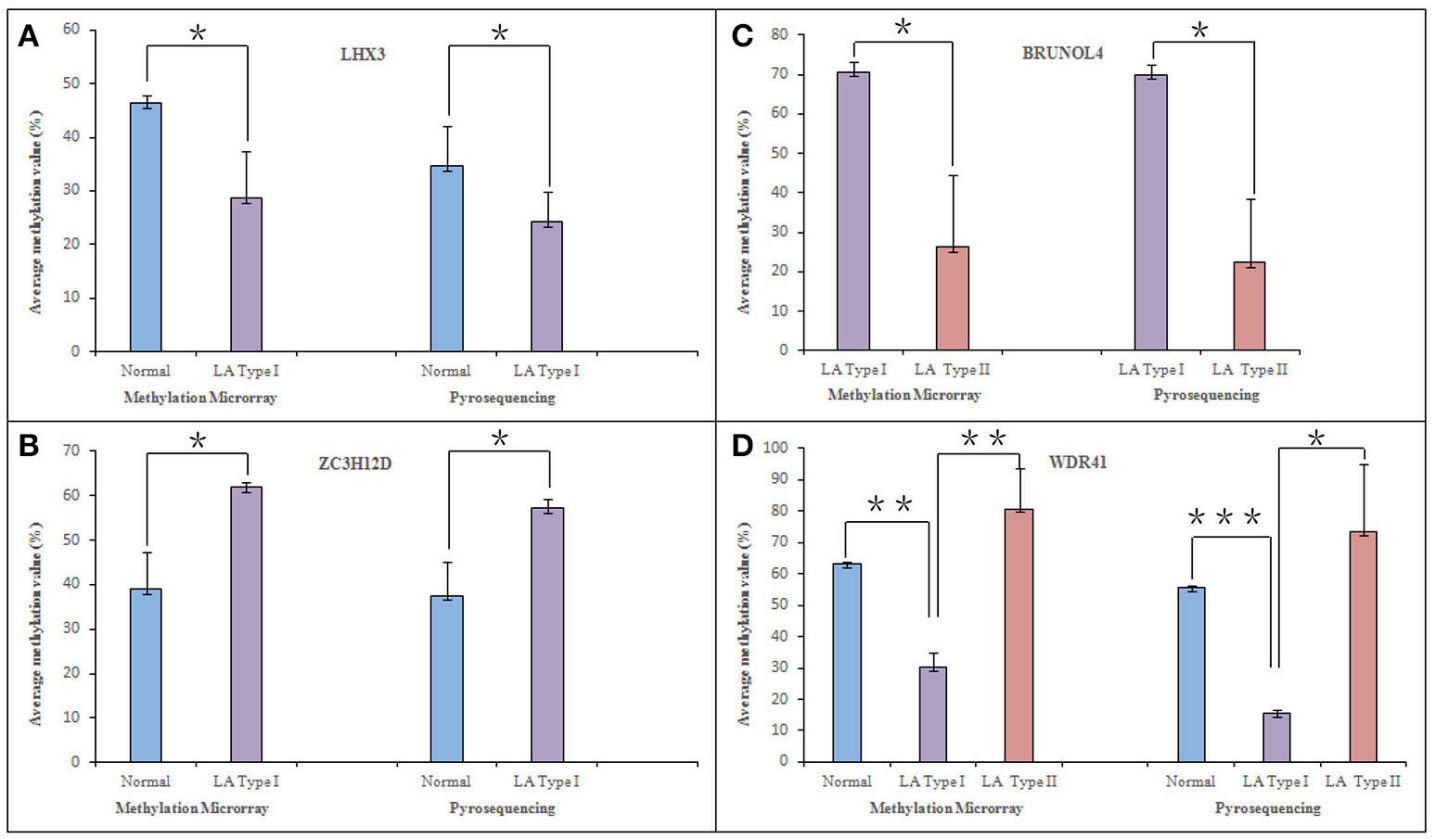
Pyrosequencing verification of hyper (hypo)-methylated CpG sites in selected genes. Four differentially methylated genes, *LHX3, ZC3H12D, BRUNOL4*, and *WDR41*, were selected from microarray analysis data and confirmed in original samples used in the methylation microarray analysis through pyrosequencing technology. They are associated with LA occurrence and (or) progression. The figure shows the comparison of methylation in *LHX3* **(A)**, *ZC3H12D* **(B)**, *BRUNOL4* **(C)**, and *WDR41* **(D)** in between pyrosequencing and microarray analysis, respectively. ^*^P < 0.05, ^**^P < 0.01, ^***^P < 0.001.

Focusing on four novel hypermethylated/hypomethylated genes (*LHX3, BRUNOL4, ZC3H12D, WDR41*) which were suggested to associate with LA occurrence or (and) progression, we confirmed the microarray data by pyrosequencing. The analysis was performed in those samples originally run on the array. As expected, all genes showed a tight correlation between the methylation values obtained using the array and the values obtained by pyrosequencing (Figure 4). Of the selected genes, *ZC3H12D* encoding a novel CCCH-zinc finger protein had significant consistence between mean methylation level detected in those subjects by DNA methylation array (LA type I vs. Normal: *n* = 3, *p* < 0.05) and that detected in independent new patients by pyrosequencing validation (LA type I vs. Normal: *n* = 12, *p* < 0.01) shown in section Integrated Analysis of DNA Methylation and Gene Expression Profiling Identifying the Inflammation-Associated *ZC3H12D* Gene in LA below. This consistency confirmed the informativeness of the single CpG sites explored in the methylation array.

### Gene-ontology analysis indicating the immune and inflammatory entity of LA

**Figure 5:**
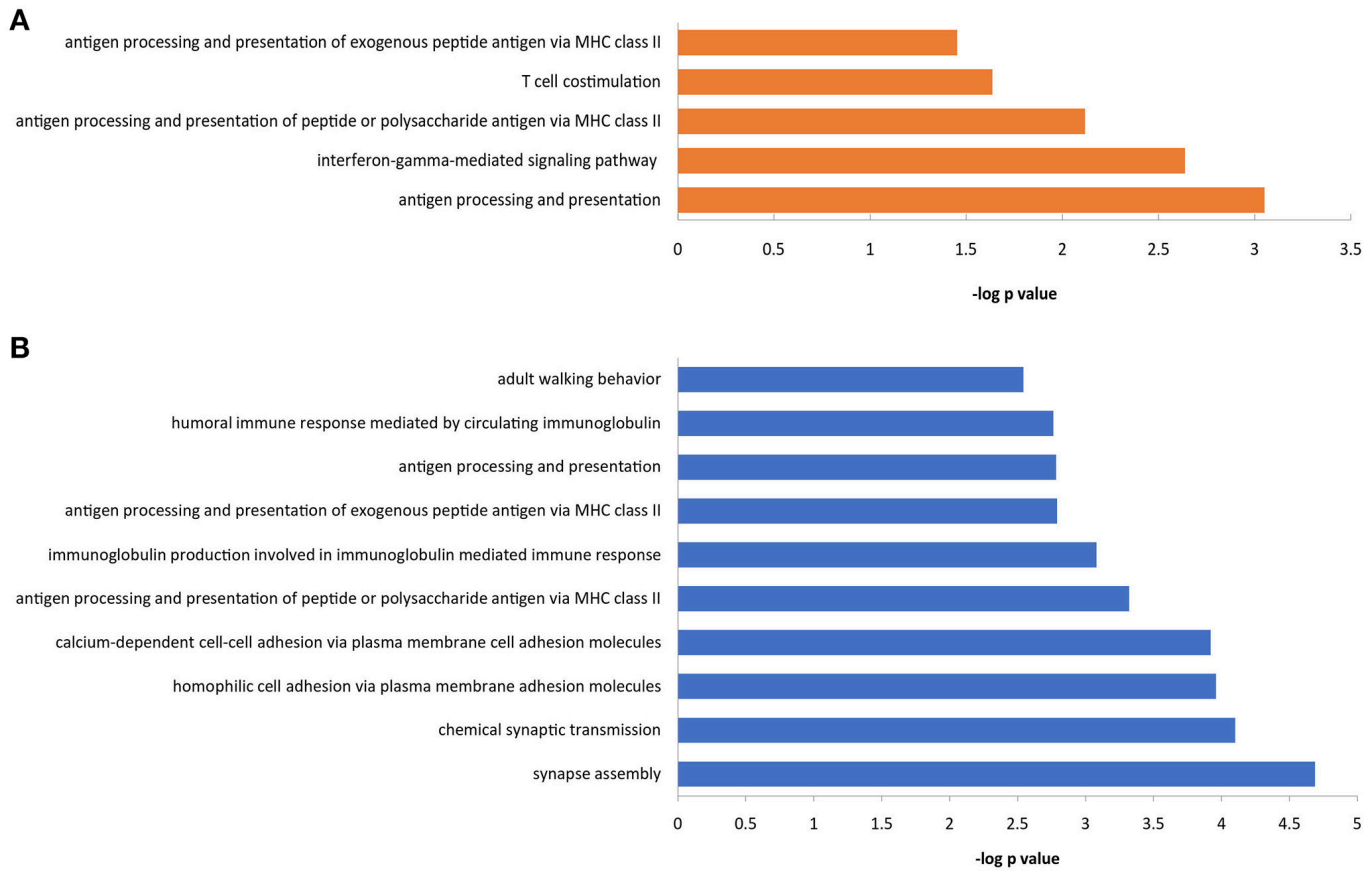
Gene ontology analysis of those LA -associated genes with aberrant methylation. Top significant biological processes enriched with the differentially methylated genes in LA occurrence **(A)** and progression **(B)** were shown, respectively.

Gene-Ontology (GO) analysis of differentially methylated genes identified by the array demonstrated a number of enriched physiological processes (Figure 5, Supplemental Table S7). GO analysis ranked by –log *p*-value revealed that many of the genes implicated both in the occurrence and progression are enriched in immune response-associated processes, such as antigen processing and presentation via MHC class II. Furthermore, differentially enriched pathways were significantly implicated in the occurrence and progression. They showed that inflammation and immune signaling, for example, antigen processing and presentation of exogenous peptide or polysaccharide antigen via MHC class II, interferon-γ-mediated signaling pathway and T cell con-stimulation all of which reached the nominal level of significance (*p* < 0.05) appeared to play a key role in the occurrence, whereas change of synapse assembly, chemical synaptic transmission, hemophilic cell adhesion and calcium-dependent cell-cell adhesion (Benjamini-corrected *p* < 0.05) which may be subsequent consequence of inflammation response seemed to contribute to the progression.

### The abnormal expression of inflammation and immune response-related genes with aberrant methylation in clinical blood samples

**Figure 6:**
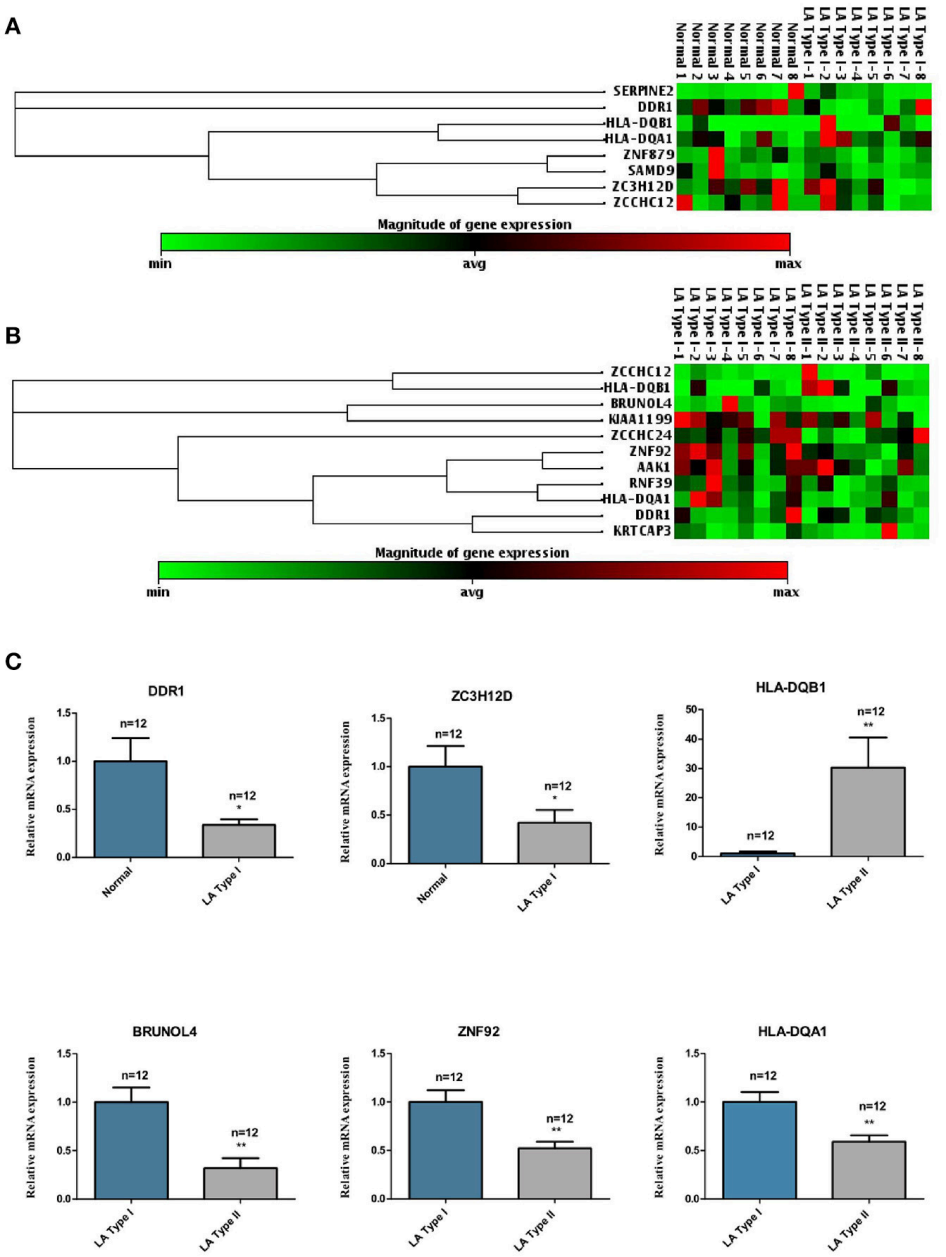
The mRNA expression levels of the LA occurrence and progression-associated genes in clinical LA samples. **(A)** represents the hierarchical clustering of 8 hyper/hypo-methylated genes associated with LA occurrence. **(B)** represents the hierarchical clustering of 11 hyper/hypo-methylated genes associated with LA progression. The green/red color represented a standardized level of mRNA expression of those genes in clinical samples. **(C)** shows the mRNA expression levels of 6 genes which were shown to be significantly up-regulated or down-regulated in LA occurrence (LA Type I vs. Normal) or progression (LA Type I vs. Normal) in additional 12 clinical LA samples. ^*^P < 0.05, ^**^P < 0.01, ^***^P < 0.001.

In order to identify those potential genes whose expression were regulated by DNA methylation in the occurrence and progression of LA, we therefore determined the expression levels of 19 candidate genes including eight occurrence-related genes and 11 progression-related genes in the first set of blood samples by qPCR (Figures 6A,B). Then, those six differentially expressed genes identified in the first set of samples were further confirmed in the second set of blood samples including 12 cases and 12 controls. As shown in the Figure 6C, both genes *DDR1* and *ZC3H12D* identified as hypermethylated on the promoter region in LA occurrence showed lower gene expression in patients with type I LA than subjects without LA. Among other four differentially expressed candidates which showed hypomethylation in the gene body in LA progression, except *HLA-DQB1* showing increased gene expression, those genes (*HLA-DQA1, ZNF92*, and *BRUNOL4*) showed lower expression in patients with type II LA than those with type I LA. Those results indicated that promoter hypermethylation of *DDR1* and *ZC3H12D*, and *HLA-DQB1* hypomethylation on the CpG island may contribute to the occurrence and progression of LA, respectively, through the regulation on the gene expression at transcription level. Since all of those three genes showing anti-correlation between DNA methylation and mRNA expression are involved in the NF-κB, TLR, TGF-β-Smad and (or) MHC receptor pathways, it further suggested that inflammation and immune response may contribute to the pathogenesis of LA, as described in GO and KEGG analysis of hyper-/hypo-methylated genes associated with LA.

### Integrated analysis of DNA methylation and gene expression profiling identifying the inflammation-associated *ZC3H12D* gene in LA

**Figure 7:**
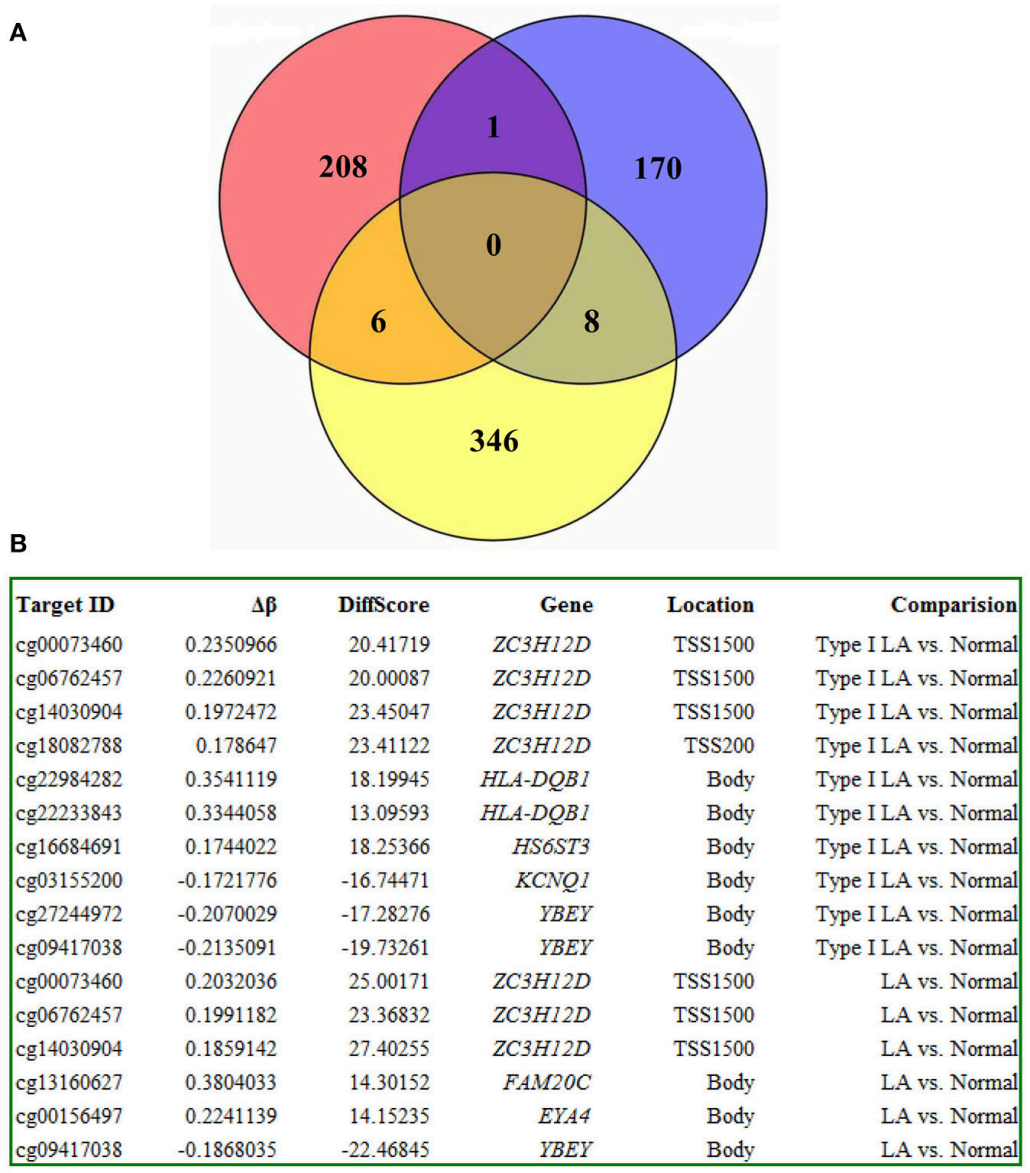
The integrated analysis on DNA methylation microarray data and gene expression microarray data of LA. **(A)** The red circle represents the differentially methylated genes (up to 215 genes) identified from the DNA methylation analysis of LA in blood. The blue circle represents those differentially expressed genes identified from the whole blood gene expression microarray analysis of LA (up to 179 genes). The yellow circle represents those differentially expressed genes identified from the gene expression microarray analysis of LA in brain tissue (up to 360 genes). The integrated analysis revealed 7 overlapping genes between DNA methylation microarray data and gene expression microarray data of LA. **(B)** The information of 7 overlapping genes about the methylation degree, differentially methylated sites, location and analysis method were shown, respectively.

**Figure 8:**
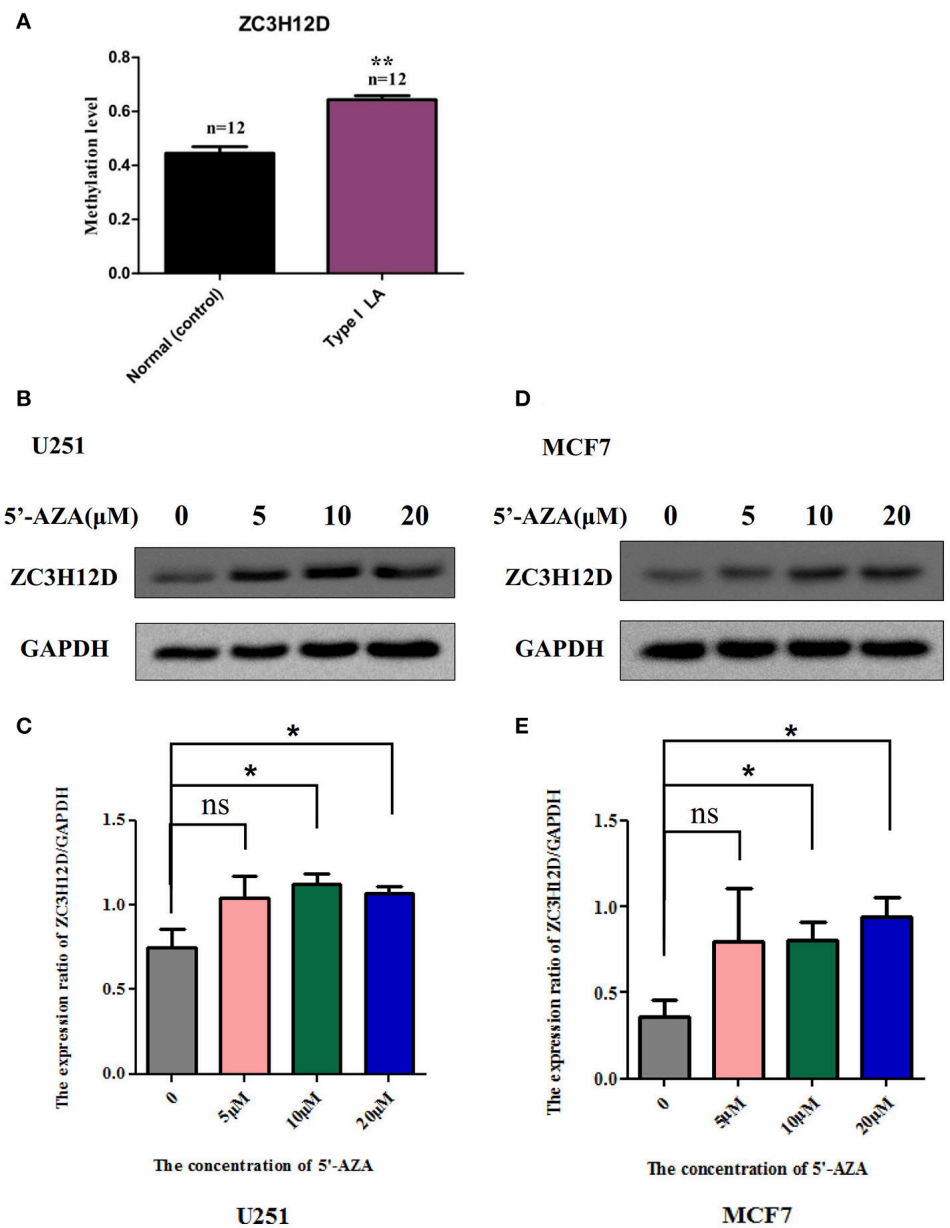
The methylation levels of *ZC3H12D* in independent clinical samples and the inhibition of *ZC3H12D* methylation on gene expression. **(A)** The methylation levels of the CpG site, cg06762457 located in *ZC3H12D* gene promoter, in 24 clinical blood samples were determined through pyrosequencing. **(B)** *ZC3H12D* expression is increased in glioma cell line U251 after treatment with different concentration of demethylation agent 5′-Aza (0, 5, 10, and 20 μM) by western blot. **(C)** Quantitative analysis of the results from three independent experiments represented in **(B)** showing that the hypermethylation in *ZC3H12D* down-regulated gene expression in U251 cell. **(D)** *ZC3H12D* expression is increased in breast tumor cell line MCF7 after treatment with different concentration of demethylation agent 5'-Aza (0, 5, 10, and 20 μM) by western blot. **(E)** Quantitative analysis of the results from three independent experiments represented in **(D)** showing that the hypermethylation in *ZC3H12D* inhibited gene expression in MCF7 cell.

To strengthen the confidence in our candidate selection, we took advantage of distinctive RNA expression profiling of LA in blood (analyzed by Xu and coworkers) and tissue (analyzed by Simpson and coworkers) obtained from two published papers. We conducted an integrated analysis of DNA methylation and gene expression profiling of LA to identify those differentially expressed genes that could be regulated by aberrant DNA methylation. As shown in Figure 7A, 6 genes identified in DNA methylation microarray analysis of LA overlapped those identified in gene expression profiling of cerebral white matter lesions, while there was only one shared gene named as *YBEY* between DNA methylation profiling and whole blood gene expression profiling. Unfortunately, and none of hyper-/hypo-methylated genes showed anti-correlation with RNA expression both in whole blood gene expression profiling and in gene expression profiling of white matter lesions in brain tissue. Among those seven differentially methylated genes with aberrant RNA expression, all of those six differentially expressed genes showed abnormal methylation just in the gene body, while *ZC3H12D* with an absolute fold change >2 showed significant methylation change on several CpG sites in the upstream of its promoter, indicating the importance of that hypermethylated gene (Figure 7B). Based on these integrated analyses, *ZC3H12D* was selected for subsequent validation for promoter methylation and mRNA expression in clinical samples by pyrosequencing and qPCR, respectively. Our results confirmed that *ZC3H12D* was hypermethylated on the promoter region and down-regulated in the patients with type I LA compared to controls without LA (Figures 6C, 8A). Those findings suggested the putative significance of *ZC3H12D* in the pathogenesis of LA.

### The regulation of promoter hypermethylation in *ZC3H12D* on the gene expression in cell lines

To explore whether reduced expression of *ZC3H12D* is associated with DNA hypermethylation on their gene promoters, we detected the change of protein expressed in two selected cell lines through the demethylation test. As shown in Figure 8 and Supplemental Figure S3, the demethylation treatment by 5′-aza-2′-deoxycytidine (5′-Aza) dramatically enhanced the expression of ZC3H12D protein in both U251 (Figures 8B,C) and MCF7 (Figures 8D,E) cell lines, suggesting that *ZC3H12D* expression could be regulated by DNA methylation. Since LA showed aberrant changes of *ZC3H12D* in DNA methylation and mRNA level, those above results thus indicated that the epigenetic regulation of *ZC3H12D* on gene regulation may be involved in the pathogenesis of LA.

## Discussion

During the past decade, few published studies showed reliable genetic component in the etiology of LA (Carmelli et al., 1998; Turner et al., 2004; Smith et al., 2009; Fornage et al., 2011). Environmental factors have been considered to contribute to human diseases through modification on those genetic factors, a phenomenon called epigenetic mechanisms (such as DNA methylation) (Iraola-Guzman et al., 2011; Grueter and Schulz, 2012). Therefore, large-scale screening of DNA methylation is helpful to reveal the epigenetic modification underlying this complex disorder.

In this study, we used the Illumina 450k DNA methylation assay to investigate DNA methylation profile of LA, and found large accounts of DNA-methylation changes in numerous loci in LA. As expected, we identified different epigenetic changes in LA subgroups, reflecting different molecular basis between the process named occurrence from Normal to LA type I, and the process named progression from LA type I to LA type II. Integrated GO analysis showed that epigenetic changes underlying the occurrence of LA were mainly enriched in immune response-associated processes (antigen processing and presentation, T cell costimulation and interferon-γ-mediated signaling pathway), whereas those changes underlying the progression process of LA were significantly enriched in synapse assembly, synaptic transmission and cell adhesion.

Overall, the majority of significantly differentially methylated genes implicated in both occurrence and progression shared proteins which were reported to be associated with inflammation and immune response-associated signaling (such as *HLA-DQA1, HLA-DQB1, HLA-DRB5, DDR1, FMOD, RAD51L1*), and nervous system functions (such as *CACNA2D4, KCNQ1, P2RX5, HCN2, KIFC2, RAB11B, WDR41, ZCCHC12*). To further understand how those differentially methylated genes lead to the LA occurrence and progression and which pathways were potentially involved in this complex pathophysiology, we also searched for large accounts of those genes-associated articles that were published until April 2018, and found that most of those genes with |Δβ|≥ 0.4 and |DiffScore|>13 also overrepresented in the following key signaling: Wnt/β-catenin, BMP/TGFβ/TGFR3, Ras-MAPK, NF-κB, JAK-STAT and Smad pathways. They included genes of *NDRG1, ZC3H12D, GIPC2, BGN, KIAA1199, CLDN4, SERPINE2, SAMD9, DDR1, GPC5, MCC, FGFB3, FGFBR3, SMOC2, and CNTN4*. For example, *BGN* encodes a member of class I family of small leucine-rich proteoglycans (SLRPs), an extracellular matrix protein that plays roles in collagen assembly, regulation of inflammation and innate immune, maintainance of synapse stability by acting as a ligand of LRP6 and MuSK, interacting with TLR2/4 receptors, activation of Smad and Runx2 signaling, and modulation of TGF-β, and BMP2/4 functions (Wegrowski et al., 1995; Nastase et al., 2012). Recently, it was reported to be enriched in the brains of subjects with cerebral autosomal dominant arteriopathy with subcortical infarcts and leukoencephalopathy (CADASIL), indicating the potential roles in white matter changes (Zhang et al., 2015). This CADASIL-enriched protein can accumulate in vessels by mTOR-mediated transcriptional activation and/or post-translational accumulation which are mediated by the protein interactions with NOTCH3 and collagen (Zhang et al., 2015). *DDR1* encodes a receptor tyrosine kinase which is ubiquitously expressed at low levels in a variety of epithelial tissues and with high levels of expression in the brain (Barbara Roig, 2010). It is activated by various types of collagens and is known to regulate cell-cell adhesion, migration, survival, and proliferation through NOTCH1, p38, STAT1/3, JAK2-STAT3 and integrin signaling pathways (Jonsson and Andersson, 2001; Barbara Roig, 2010; Chetoui et al., 2011; Kim et al., 2011; Yeh et al., 2011; Gao et al., 2016). *SAMD9* encodes a sterile alpha motif domain-containing protein that interacts with RGL2 to diminish the expression of EGR1 and regulate the inflammation in response to the inflammatory cytokines including TNF-α and IFN-γ (Hershkovitz et al., 2011). In addition, contactin 4 encoded by the *CNTN4* gene, is an axon-associated cell-adhesion molecular highly expressed in the brain, which may play an essential role in the formation, maintenance and plasticity of neuronal network (Shimoda and Watanabe, 2009; Bouyain and Watkins, 2010). These data indicated the putative inflammatory process underlying the demyelination pathology of LA. According to those epigenetic data, we suggested that immune and inflammation played an essential role in the early disease and further contributed to the severe lesions through disturbing normal neuron behaviors (synaptic assembling and synaptic transmission) and cell performance (such as cell adhesion) in the late stage of neuroinflammatory disease.

In our methylation data, there were limited overlapping genes with those identified in the Genome-Wide Association Studies (GWAS) and RNA expression analysis of LA (Simpson et al., 2009; Xu et al., 2010; Fornage et al., 2011). However, it's plausible that, similar to the enrichment results of differentially methylated genes identified in our study, data from GWAS and RNA expression profile of LA also illustrated the crucial role of immune and inflammation in the development of LA (Simpson et al., 2009; Xu et al., 2010; Fornage et al., 2011). In the GWAS of LA from European descent, 6 novel single nucleotide polymorphisms (SNPs) in the Chr17q25 locus including *TRIM65, TRIM47* and *WBP2*, were identified to be risk factors of LA. Those risk genes were related with immune regulation, inflammatory signaling, and cell apoptosis. TRIM65 and TRIM47, two members of tripartite motif-containing (TRIM) proteins superfamily, expressed in response to INFs, and participated in innate immunity, cell cycle regulation, apoptosis, vesicular trafficking and neuroprotection. They were shown to be associated many human diseases, such as some genetic disorders, autoimmune disease, cancers, neurological disorders (Ozato et al., 2008; Wang et al., 2016; Han et al., 2017). WBP-2, a putative ligand that binds to the WW-domain of Yes kinase-associated protein 1(YAP1) with relatively high affinity and specificity through PPXY motifs, acts as a coactivator of gene transcription regulation (e.g., estrogen receptor (ER)/ progesterone receptor (PR) transactivation pathway) and it may play important roles in diverse pathological conditions, such as cancer, inherited genetic diseases, and inflammation (Chan et al., 2011; Chen et al., 2017). Microarray RNA expression analysis in both blood and brain tissue from subjects with LA identified multiple WMH-specific genes with altered RNA expression. Those up-/down-regulated genes were reported to be associated with immune and inflammation, oxidative stress, ion and electron transport, hormone signaling, and cell performance (cell cycle, apoptosis and metabolism), as well as neuron system behaviors (oligodendrocyte proliferation, axon repair, long-term potentiation, and neurotransmission) (Simpson et al., 2009; Xu et al., 2010), providing additional evidence for the implication of inflammation and immune response in LA pathogenesis.

Among those limited overlapping genes between DNA methylation profiling and expression profiling of LA, *ZC3H12D* was selected to study together with other candidate genes. Among those differentially expressed genes, three significantly hypomethylated genes including *BRUNOL4, ZNF92*, and *HLA-DQA1* didn't showed negative correlation but positive correlation between DNA methylation level and mRNA expression. This inconsistence may be due to the fact that the significantly hypomethylated CpG sites of those genes were not located in the promoter region, but in the gene body. Methylation in gene body has also been considered to play roles in the regulation of gene transcription either through suppressing the unwanted expression of non-coding RNAs and the expression of transposons or through regulating the use of alternative promoters(Maunakea et al., 2010; Shenker and Flanagan, 2012). However, our limited results could not confirm this opinion. It is necessary to verify the rationality of inclusion of CpG sites located in gene body in Illumina 450K methylation array, and investigate their functions in the regulation of gene expression in large scale of gene screening in future. On the contrary, other three genes which were differentially methylated in CpG sites located in the promoter region or CpG island, including *DDR1, HLA-DQB1*, and *ZC3H12D*, showed negative correlation between DNA methylation level and mRNA expression in accordance with our expect, indicating the potential functions of those genes in LA. In particular, this regulation of DNA promoter methylation on *ZC3H12D* expression confirmed by biochemistry experiment in cell lines may provide an important clue for its potential role in the pathogenesis of LA. *ZC3H12D* encodes the fourth member of a novel CCCH-zinc finger protein family which comprises of MCPIP1, 2, 3, and 4 (Zhou et al., 2006; Liang et al., 2008). It was originally reported as a putative tumor suppressor because of its down regulation in transformed follicular lymphoma (Minagawa et al., 2007). Recently, ZC3H12D was suggested as a novel negative feedback regulator of TLR signaling and macrophage activation (Liang et al., 2008; Huang et al., 2012). It was shown that the expression of ZC3H12D was remarkably induced by TLR ligands through JNK and NF-κB signal pathways in macrophages. However, overexpression of ZC3H12D significantly inhibited TLR2 and TLR4 activation-induced JNK, ERK and NF-κB signaling as well as macrophage inflammation (Liang et al., 2008; Huang et al., 2012). These findings suggested that it may play an important role in host immunity and inflammatory diseases (Liang et al., 2008). According to the changes of *ZC3H12D* in LA and its function in inflammation, we here hypothesized that this gene may suppress its expression through promoter methylation to alleviate its inhibition on immune and inflammation response, thus promoting the inflammatory pathogenesis of LA.

Together, it seems that inflammatory pathways induced or regulated by those genes with the changes in methylation and expression, such as *ZC3H12D, HLA-DQB1* and *HLA-DQA1* are strongly involved in the pathogenesis of this complex disorder. This concept could be supported by the previous biochemistry data that inflammatory molecules including lipoprotein-associated phospholipase A2, myeloperoxidase, C-reactive protein, and IL-6, were shown to be increased in the blood of LA subjects (Pantoni, 2002).

White matter is composed of millions of bundles of axons coated by the unique, compact myelin sheaths (Fields, 2010). Myelin is produced by oligodendrocytes, which wrap up to 150 layers of tightly compressed cell membrane around axons (Fields, 2010). Acting as the electrical insulation, myelin is required for maximizing the conduction velocity of nerve impulses and it is essential for normal brain function. Its damage can disrupt conduction and consequently impair sensory, motor, and cognitive functions (Fields, 2010). Pathologically, LA is mainly characterized by loss of myelin and axons, and patchy demyelination in the cerebral white matter lesions of patients with LA. Demyelination injury, failure of remyelination by oligodendrocytes, and unsuccessful myelin repair which were shown to be associated with other demyelination diseases, such as multiple sclerosis (MS) and leukodystrophies, may be the cause of pathological changes in LA (Trapp et al., 1998; Mar and Noetzel, 2010). Published papers about CNS demyelination injury have showed that multiple inhibitors or regulators, such as Jagged 1 (a ligand of the Notch1 receptor), Lingo-1 (a transmembrane protein containing a leucine-rich repeat and an immunoglobulin domain), PSA-NCAM (polysialic acid–containing neural cell adhesion molecule), control oligodendrocyte differentiation and the myelination of individual axons. Failure of myelin regeneration arises from abnormal activation of negative regulatory pathways, such as bone morphogenetic protein (BMP), Wnt signaling, and Notch signaling (Miller and Mi, 2007). Those hyper-/hypo-methylated genes identified from the occurrence (LA type I vs. Normal) (e.g., *BGN, TGFBR3*), the progression (LA type II vs. type I) (e.g., *GPC5, CLDN4, FGFB3*), and potentially third progress (LA type II vs. Normal) (e.g., *KIAA1199, AAK1, MCC*) are associated with Wnt-β-catenin, BMP-TGFR3, Smad and (or) Notch signaling (Wegrowski et al., 1995; Jonsson and Andersson, 2001; Zakrzewicz et al., 2007; Birkenkamp-Demtroder et al., 2011; Kim et al., 2011; Li and Yang, 2011; Liu et al., 2012; Nastase et al., 2012; Townsend et al., 2012; Li et al., 2013), suggesting that those genes including *ZC3H12D* with aberrant DNA methylation may result in the failure of remyelination and subsequent demyelination through direct or indirect regulation on those inflammation-associated pathways.

**Figure 9:**
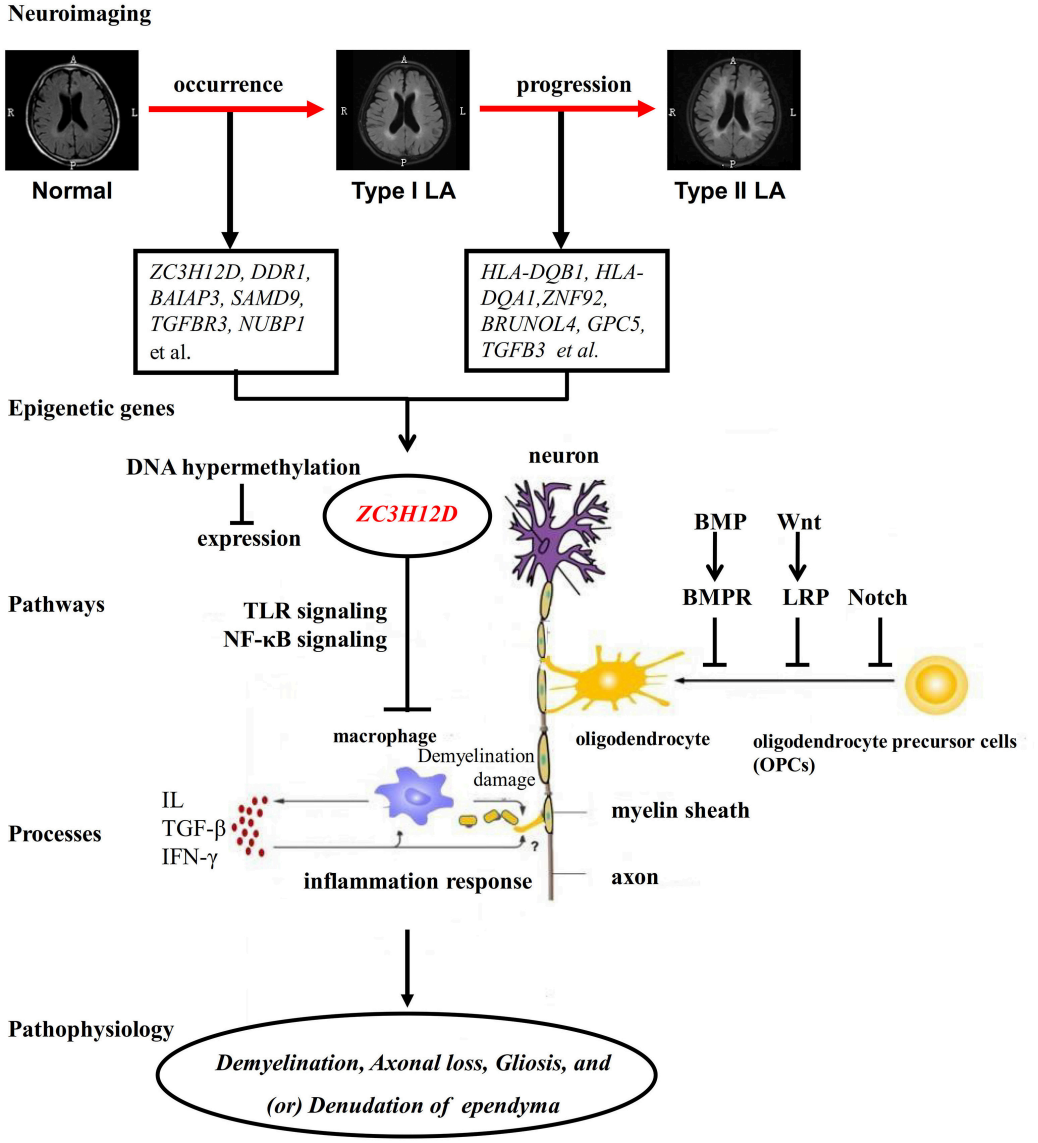
The putative pathogenesis of LA. LA seems to be a progressive disorder which includes two processes. The process from Normal to Type I LA and that from Type I LA to Type II LA represent the occurrence and progression of LA, respectively. LA is always considered as a complex illness which is driven by both genetic and environmental factors. Large accounts of differentially methylated genes are implicated in those two processes of LA. Among these genes with aberrant methylation, *ZC3H12D* showed hypermethylation on the promoter region and reduced expression in the occurrence of LA. As an inflammation-associated gene, *ZC3H12D* could inhibit the inflammatory process in macrophage through the TLR and NF-κB signaling. The inhibition of ZC3H12D on those above pathways and macrophage inflammation is relieved through the decreased expression resulted from the promoter hypermethylation. These finally lead to the macrophage activation, subsequently promote downstream inflammation response and result in demyelination damage on myelin in white matter. The activated inflammation response also may inhibit the oligodendrocyte differentiation, maturation and remyelination through unknown pathways. On the other hand, the occurrence and progression-related genes with aberrant DNA methylation could directly regulate those above physiological processes through their regulation on those negative pathways (BMP, Wnt and Notch signaling) of myelination. Finally, the neuroinflammation induced by the activation of those upstream genes and pathways in response to pathological stimulation may act as the downstream mechanism to disturb the balance of myelination and demyelination, leading to pathological characteristics in brain, such as patchy demyelination, axonal loss, gliosis, and denudation of the ependyma, and subsequent LA showing white matter hyperintensities on FLAIR-MRI.

As described above, consistent with enrichment results of functional pathways from DNA methylation analysis, those findings from RNA expression and SNP studies strongly illustrated the role of inflammation in LA pathogenesis. Here, according to the considerable clinical, epidemiological and genetic evidence, we hypothesized that extrinsic and intrinsic factors may drive the cellular signaling pathways (Wnt/β-catenin, BMP/TGFβ/TGFR3, Ras-MAPK, NF-κB, JNK, JAK-STAT, TLR and (or) Smad pathways) to trigger the inflammation processes (antigen processing and presentation, T-cell co-stimulation, and (or) interferon-γ-mediated signaling, resulting in local myelin damage of white matter in the early LA, and aggravated inflammation effect impaired neuron functions and induced demyelination injury and the failure of oligodendrocyte differentiation, maturation and remyelination, and further led to large regions of white matter lesions with severe demyelination, abnormal neuron synaptic transmission/assembling and cell adhesion (neuron axons-oligodendrocytes interaction) and other unknown molecular and cell changes in the late LA (Figure 9). The change of inflammation-associated gene *ZC3H12D* in promoter methylation and expression implicated in LA may drive or regulate those inflammatory processes in macrophage through those above signaling pathways (TLR, NF-κB et al.) and contributed to the subsequent demyelination injury on myelin in white matter (Figure 9).

The above hypothesis was not only supported by the recent transcriptome-wide association study reporting that 13 genes most of which are involved in inflammation-related pathways were significantly associated with WMH (Lin et al., 2017), but also by previous studies reporting that chronic ischemia, endothelial dysfunction and blood–brain barrier breakdown played an important role in the pathogenesis of LA (Pantoni and Garcia, 1997; Pantoni, 2002), and inflammation response may be the cause of or the result of the endothelial dysfunction and blood–brain barrier (BBB) breakdown in WMH subjects (Fornage et al., 2008). Although the crucial factors for inflammation response and the relationship of inflammation with endothelial dysfunction and blood–brain barrier in the development of LA are poorly understood to date, it's inspiring to indicate the potential inflammation mechanism underlying the white matter lesions. Further work is required to solve the questions by functional studies in the animal model of LA.

To our knowledge, in this study, we for the first time attempted to characterize the genome-wide methylation of LA in Chinese population and identified the inflammation-associated *ZC3H12D* implicated in LA. However, it also has some limitations: 1) its small sample size, future studies on larger cohorts will be needed to replicate the results and further search more epigenetic markers in LA, 2) no time course involved in this study, the different epigenetic mechanisms between the occurrence and progression through screening differentially methylated genes among three groups of subjects were not synchronized. Moreover, epigenome of complex disorder might change throughout the lifetime of an individual. Therefore, this study just represented a picture of LA methylation profile at a single time point. 3) DNA was not isolated from brain tissue, but blood in subjects with LA. Although gene expression changes which could be regulated by DNA methylation in the blood are considered to mirror those in the brain (Achiron and Gurevich, 2006; Sullivan et al., 2006), more studies are necessary to confirm the potential functions of those epigenetic genes identified from whole blood in cerebral white matter and WMLs. 4) The precise role of *ZC3H12D* in LA has not been assessed in animal model. Therefore, further studies are needed to elucidate the putative mechanisms of LA.

## Conclusions

Our study for the first time provided the whole genome-scale DNA methylation analysis of LA in Chinese population, and revealed that extensive DNA methylation was allied with both occurrence and progression of LA. Together with the fact that most of differentially methylated genes and those enriched pathways were strongly associated with inflammatory and immune response, synapse assembly and transmission and cell adhesion, the finding that inflammation-associated gene *ZC3H12D* with significant change in promoter methylation and expression was implicated in LA suggested LA as a CNS neuroinflammatory white matter illness. Our findings here have clinical implications on LA diagnosis, and it could not only light up the new direction of LA pathogenesis but also provide the potential of LA treatment.

## Author contributions

W-QH carried out genomic DNA extraction, participated in methylation microarray analysis and pyrosequencing validation and article drafting, as well as revision; K-HY performed the quantitative real time PCR for those genes; ZL and L-LC confirmed the results of demethylation test; M-LL revised the GO analysis results. HW and H-NL polished the tables and figures; QL performed the clinical evaluation, neuropsychological examination and the assessment of brain white matter lesions by FLAIR-MRI; C-MT took responsibility in experimental design and data interpretation.

### Conflict of interest statement

The authors declare that the research was conducted in the absence of any commercial or financial relationships that could be construed as a potential conflict of interest.

## Supplementary material

Supplemental Figure S1 Distribution of differentially methylated locus in LA. **(A)** The numbers of those differentially methylated loci by chromosome in LA. **(B)** Proportion of differentially methylated loci given by island functional categories (CpG islands, shores, shelf) showing significant change of DNA methylation in LA. **(C)** Proportion of differentially methylated loci given by gene functional categories (within 200 or 1,500 bp of a transcription start site (TSS), 5′UTR, 3′UTR, gene body). **(D)** The numbers of hypermethylated and hypomethylated loci in LA. **(E)** The numbers of hypermethylated and hypomethylated genes in LA.

Supplemental Figure S2 Distribution of differentially methylated locus between occurrence and progression of LA. **(A)** Numbers of differentially methylated loci by chromosome in LA occurrence (light blue) and progression (violet) processes: the numbers of hypermethylated loci displayed on the positive y-axis and hypomethylated loci displayed on the negative y-axis. **(B,C)** Proportion of differentially methylated loci in the LA occurrence **(B)** and progression **(C)**. Bar plots of the proportion of hypermethylated loci (red) and hypomethylated loci (light green) relative to total differentially methylated loci (blue) found in CpG islands and CpG island shores. **(D)** Comparison of proportion of differentially methylated loci in LA occurrence (orange) with that in LA progression (light orange). **(E)** The numbers of occurrence (blue)/progression (red)-associated differentially methylated loci located in different gene functional regions (such as TSS 200 and TSS 1,500, 5′ UTR, the first exon of gene (1st exon), 3′UTR, gene body).

Supplemental Figure S3 Demethylation test of hypermethylated gene *ZC3H12D* in cell lines.

Supplemental Table S1 PCR and sequencing primers for pyrosequencing.

Supplemental Table S2 Hyper- and hypo-methylated loci and genes in LA.

Supplemental Table S3 Differentially methylated loci and genes in LA occurrence.

Supplemental Table S4 Differentially methylated loci and genes in LA progression.

Supplemental Table S5 Shared genes between LA occurrence and progression.

Supplemental Table S6 Differentially methylated loci and genes between subjects with type II LA and healthy controls without LA.

Supplemental Table S7 Enriched pathways implicated in occurrence and progression, respectively.

## Abbreviations

3′UTR: 3′ untranslated region
5-AZA: 5′-aza-2′-deoxycytidine
5′UTR: 5′ untranslated region
BBB: blood–brain barrier
BMP: bone morphogenetic protein
CE: capillary electrophoresis
CNS: central nervous system
DWMLs: deep/subcortical white matter lesions
ER: estrogen receptor
FLAIR-MRI: fluid-attenuated inversion recovery-magnetic resonance imaging
GO: Gene Ontology
GWAS: Genome-Wide Association Studies
LA: leukoaraiosis
MRI: magnetic resonance imaging
MS: multiple sclerosis
PR: progesterone receptor
PSA-NCAM: polysialic acid–containing neural cell adhesion molecule
PVWMLs: periventricular white matter lesions
qPCR: Quantitative real time PCR
SNP: single nucleotide polymorphisms
SLRPs: small leucine-rich proteoglycans
TRIM: tripartite motif-containing proteins
WMH: white matter hyperintensities
WML: white matter lesions
YAP1: Yes kinase-associated protein 1.
