# Supplementary figures and images for: DNA Methylation Profiling Reveals the Change of Inflammation-Associated ZC3H12D in Leukoaraiosis
Source: Front Aging Neurosci. 2018 May 23;10:143. doi: 10.3389/fnagi.2018.00143 (PMC5974056; doi:10.3389/fnagi.2018.00143)

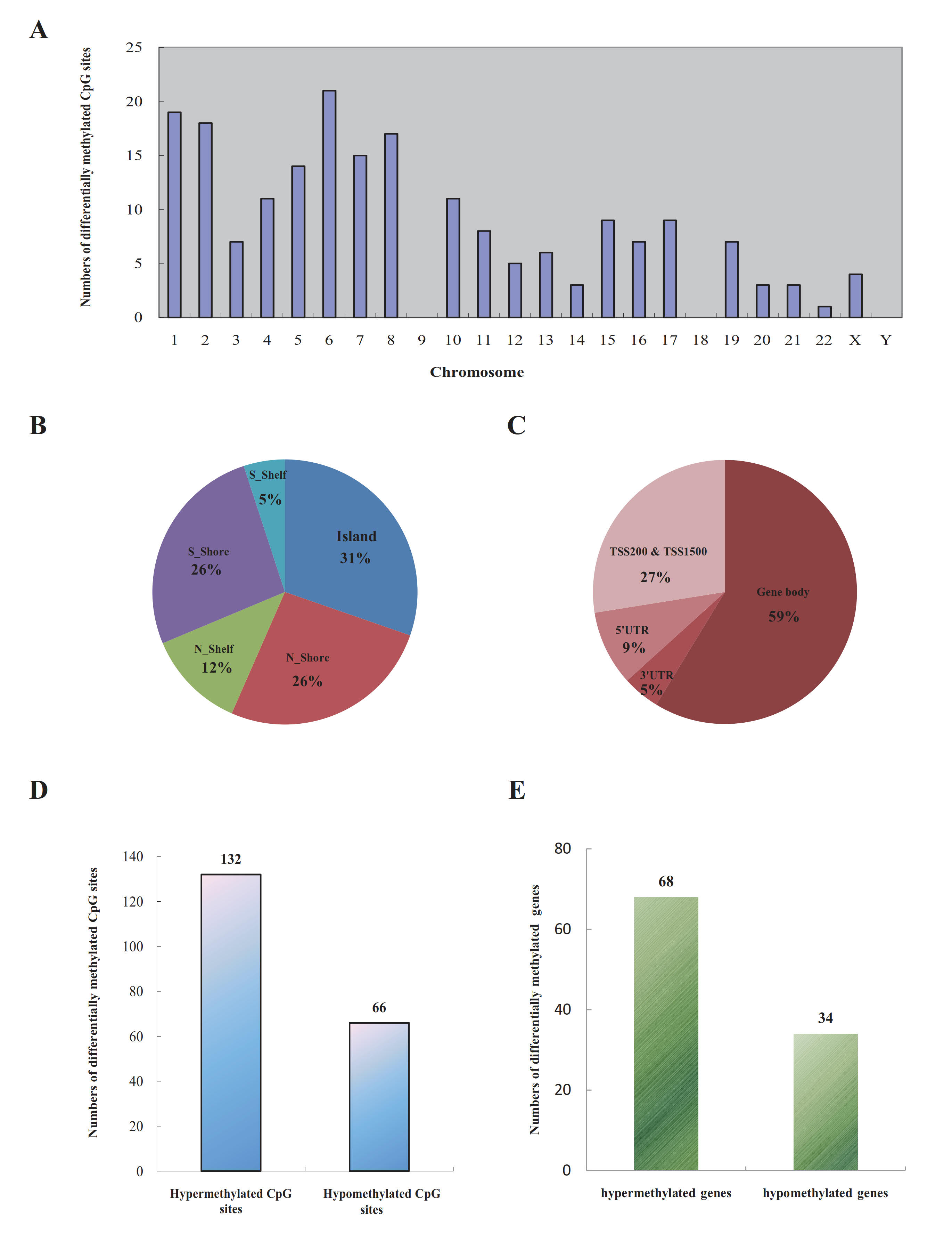

Supplement: Supplemental Figure S1 — Distribution of differentially methylated locus in LA. (A) The numbers of those differentially methylated loci by chromosome in LA. (B) Proportion of differentially methylated loci given by island functional categories (CpG islands, shores, shelf) showing significant change of DNA methylation in LA. (C) Proportion of differentially methylated loci given by gene functional categories (within 200 or 1,500 bp of a transcription start site (TSS), 5′UTR, 3′UTR, gene body). (D) The numbers of hypermethylated and hypomethylated loci in LA. (E) The numbers of hypermethylated and hypomethylated genes in LA. [file Image_1.JPEG]

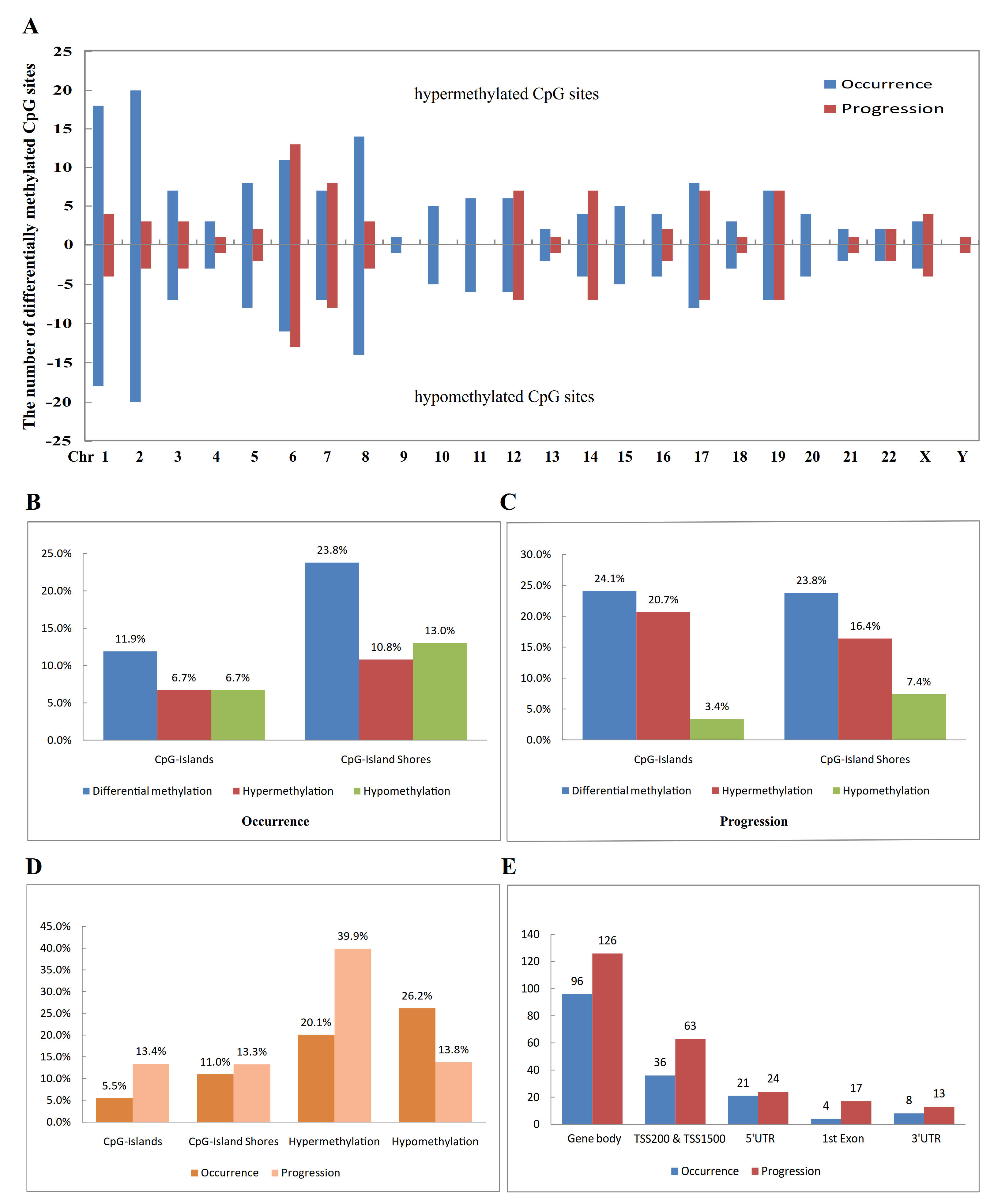

Supplement: Supplemental Figure S2 — Distribution of differentially methylated locus between occurrence and progression of LA. (A) Numbers of differentially methylated loci by chromosome in LA occurrence (light blue) and progression (violet) processes: the numbers of hypermethylated loci displayed on the positive y-axis and hypomethylated loci displayed on the negative y-axis. (B,C) Proportion of differentially methylated loci in the LA occurrence (B) and progression (C). Bar plots of the proportion of hypermethylated loci (red) and hypomethylated loci (light green) relative to total differentially methylated loci (blue) found in CpG islands and CpG island shores. (D) Comparison of proportion of differentially methylated loci in LA occurrence (orange) with that in LA progression (light orange). (E) The numbers of occurrence (blue)/progression (red)-associated differentially methylated loci located in different gene functional regions (such as TSS 200 and TSS 1,500, 5′ UTR, the first exon of gene (1st exon), 3′UTR, gene body). [file Image_2.JPEG]
