# Supplementary material for: DNA Methylation Profiling Reveals the Change of Inflammation-Associated ZC3H12D in Leukoaraiosis
Source: Front Aging Neurosci. 2018 May 23;10:143. doi: 10.3389/fnagi.2018.00143 (PMC5974056; doi:10.3389/fnagi.2018.00143)
Supplement: Supplemental Figure S3 — Demethylation test of hypermethylated gene ZC3H12D in cell lines. [file Image_3.PDF]

Figure S3. A. Demethylation test of hypermethylated gene *ZC3H12D* in U251 cell line

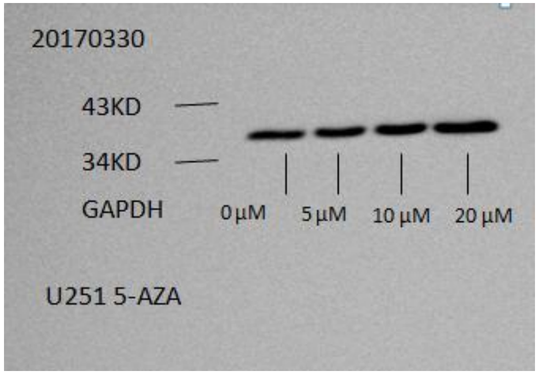


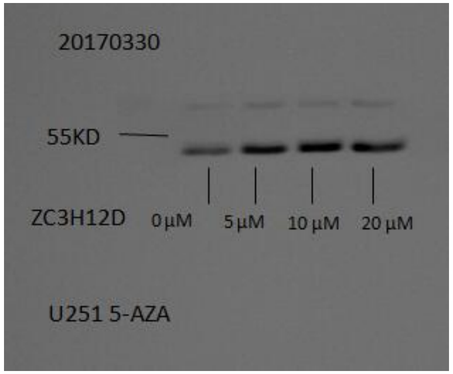


**First experiment**

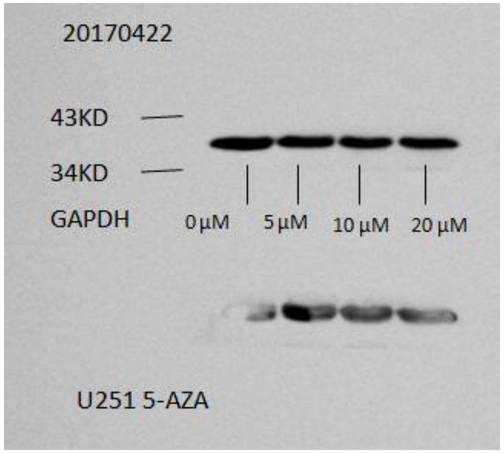


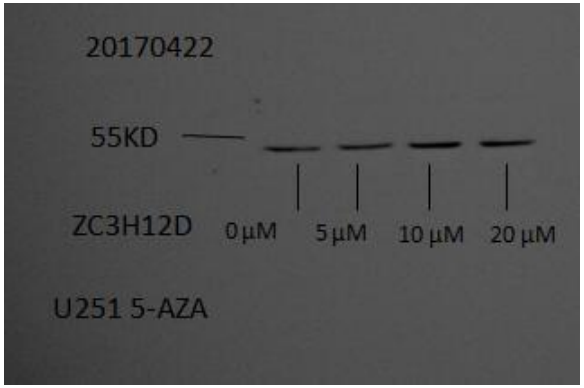


**Second experiment**

**Third experiment**

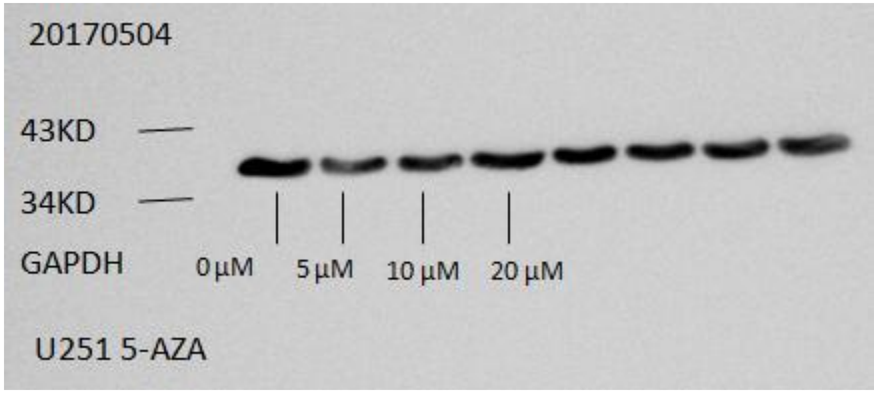


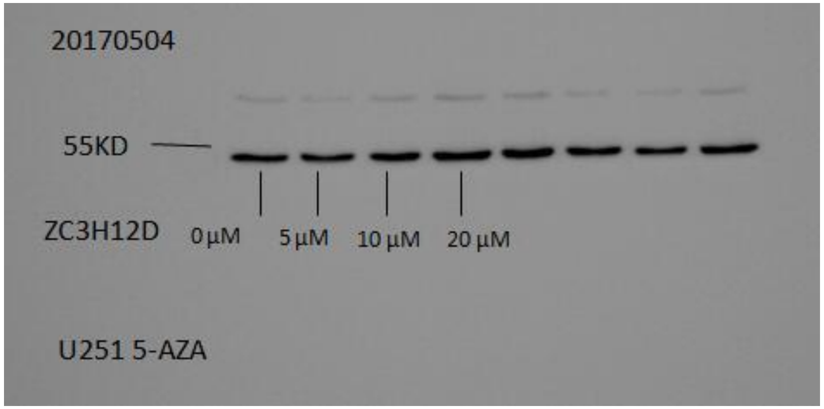

Figure S3. B. Demethylation test of hypermethylated gene *ZC3H12D* in MCF7 cell line

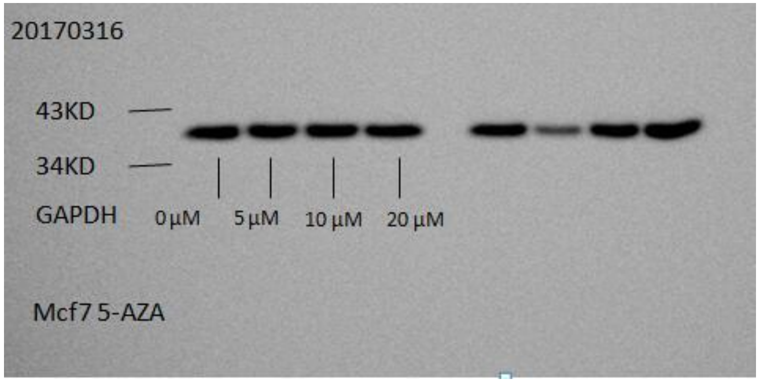


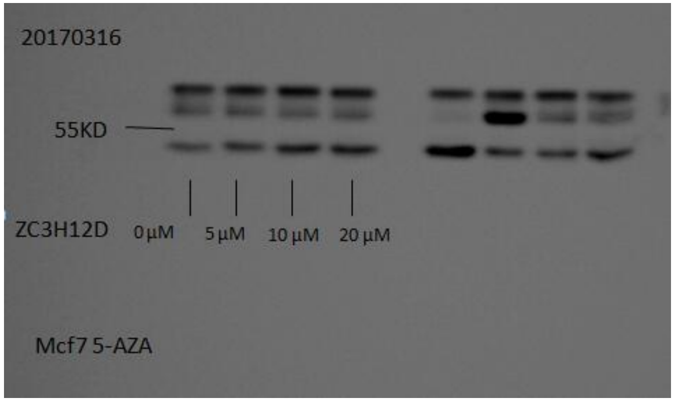


**First experiment**

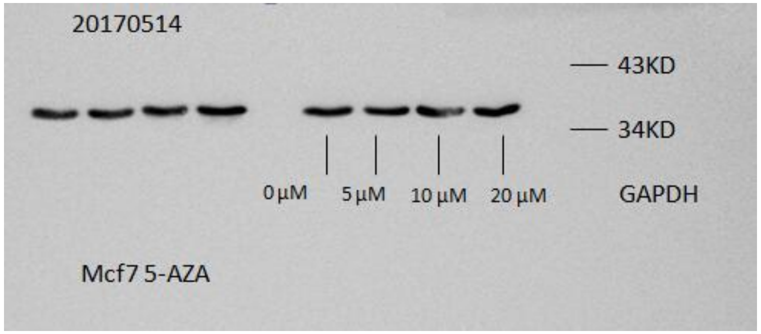


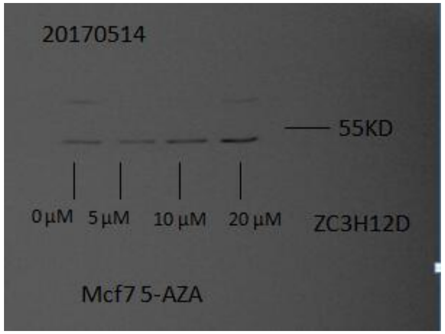


**Second experiment**

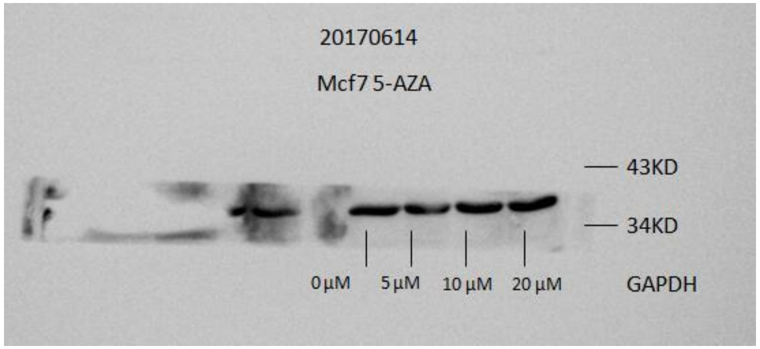


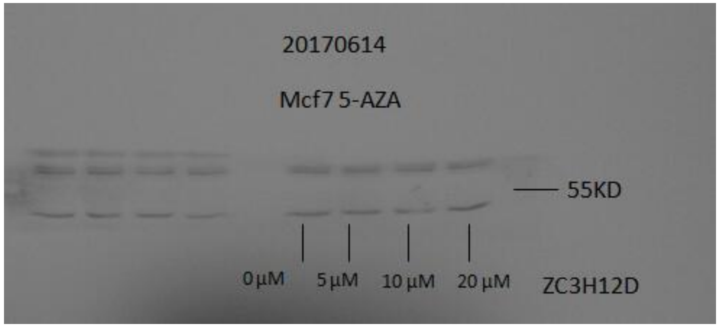


**Third experiment**
