# Supplementary material for: DNA Methylation Profiling Reveals the Change of Inflammation-Associated ZC3H12D in Leukoaraiosis
Source: Front Aging Neurosci. 2018 May 23;10:143. doi: 10.3389/fnagi.2018.00143 (PMC5974056; doi:10.3389/fnagi.2018.00143)
Supplement: Supplemental Table S1 — PCR and sequencing primers for pyrosequencing. [file Table_1.DOC]

| **CpG sites** | **Genes** | **PCR primers** | **Sequencing primers** | **Product**  **length** |
| --- | --- | --- | --- | --- |
| cg14277923 | *LHX3* | F1: 5’ GGGTTGGAGAGAGGTAAG 3’  R1: 5’ CCAAACTAACCACCACCCTACA 3’ | 5’ AGGAGGTTTAGGAGGAAGGTT 3’ | 85bp |
| cg18558969 | *BRUNOL4* | F1: 5’ATGTAGGTTTGTGTGGAGATAAT 3’  R1: 5’ ACACCCCCACCAAAAAAAAAAACAAAAATA 3’ | 5’ GTTTGTGTGGAGATAATG 3’ | 88bp |
| cg25420101 | *WDR41* | F1: 5’ GGAATATAATTTTATGGTATATATTTGAGA 3’  R1: 5’ CCACATTTTAACTAAAAAACATACATTCTA 3’ | 5’ ATTTAAAAATACTAAAATTCATAAT 3’ | 301bp |
| cg06762457 | *ZC3H12D* | F1: 5’ AAATATAGTTTGTAGGAGGAAGAGTGTTA 3’  R1: 5’ CAATAAAAAACCACAAAACCATATTATCTC 3’ | 5’ GGAGGATTTTGTTTTTGTTTTTA 3’ | 209bp |

**Supplemental Tables**

**Table S1. PCR and sequencing primers for pyrosequencing.**
